# Novel *N′*-substituted benzylidene benzohydrazides linked to 1,2,3-triazoles: potent α-glucosidase inhibitors

**DOI:** 10.1038/s41598-023-36046-y

**Published:** 2023-06-02

**Authors:** Mina Saeedi, Roshanak Hariri, Aida Iraji, Ali Ahmadi, Somayeh Mojtabavi, Shiva Golshani, Mohammad Ali Faramarzi, Tahmineh Akbarzadeh

**Affiliations:** 1 Medicinal Plants Research Center, Faculty of Pharmacy, Tehran University of Medical Sciences, Tehran, Iran; 2 Persian Medicine and Pharmacy Research Center, Tehran University of Medical Sciences, Tehran, Iran; 3 Department of Medicinal Chemistry, Faculty of Pharmacy, Tehran University of Medical Sciences, Tehran, Iran; 4 Stem Cells Technology Research Center, Shiraz University of Medical Sciences, Shiraz, Iran; 5 Central Research Laboratory, Shiraz University of Medical Sciences, Shiraz, Iran; 6 Department of Pharmaceutical Biotechnology, Faculty of Pharmacy, Tehran University of Medical Sciences, P.O. Box 14155-6451, Tehran, 1417614411 Iran

**Keywords:** Medicinal chemistry, Organic chemistry

## Abstract

Herein, various *N′*-substituted benzylidene benzohydrazide-1,2,3-triazoles were designed, synthesized, and screened for their inhibitory activity toward α-glucosidase. The structure of derivatives was confirmed using ^1^H- and ^13^C-NMR, FTIR, Mass spectrometry, and elemental analysis. All derivatives exhibited good inhibition with IC_50_ values in the range of 0.01 to 648.90 µM, compared with acarbose as the positive control (IC_50_ = 752.10 µM). Among them, compounds **7a** and **7h** showed significant potency with IC_50_ values of 0.02 and 0.01 µM, respectively. The kinetic study revealed that they are noncompetitive inhibitors toward α-glucosidase. Also, fluorescence quenching was used to investigate the interaction of three inhibitors **7a**, **7d**, and **7h,** with α-glucosidase. Accordingly, the binding constants, the number of binding sites, and values of thermodynamic parameters were determined for the interaction of candidate compounds toward the enzyme. Finally, the in silico cavity detection plus molecular docking was performed to find the allosteric site and key interactions between synthesized compounds and the target enzyme.

## Introduction

Diabetes mellitus (DM) is one of the major health challenges of the twenty-first century, characterized by chronic hyperglycemia^1^. The disease is associated with many complications, including neuropathy, nephropathy, retinopathy, cardiovascular disease, kidney disease, and liver disease as well as skin complications which result in a heavy economic and social burden^2–4^. Type-2 diabetes (T2DM) known as the most common type of DM, takes up 90% of all diabetes cases resulting from pancreatic *β*-cell dysfunction and resistance to insulin action in peripheral tissues (muscle and adipose)^5,6^. α-Glucosidase (EC 3.2.1.20) is an important hydrolase enzyme present on the surface of the small intestine. It catalyzes the hydrolysis of carbohydrates into absorbable glucose monomers by cleaving the bond between glucosidic oxygen and glucosyl residues of carbohydrates^7,8^. The inhibition of α-glucosidase delays the production of glucose monomers leading to the reduction of postprandial blood glucose levels, which is an effective approach to the management strategy of T2DM^9^. To date, a large number of α-glucosidase inhibitors have been introduced from natural origin or chemical routes^10–14^. Amongst just acarbose, voglibose, and miglitol enter the market, which is associated with side effects, especially gastrointestinal effects and hepatotoxicity^15,16^. Thus, the design and synthesis of potent α-glucosidase inhibitors is an attractive subject among researchers.

**Figure 1:**
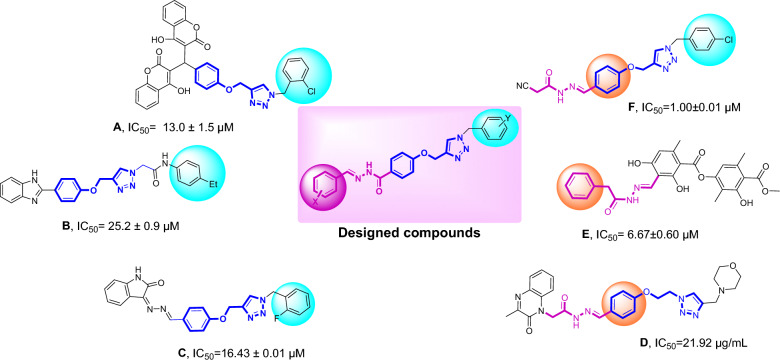
Representative inhibitors of α-glucosidase and newly designed compounds.

1,2,3-Triazole and its derivatives are the key skeletons possessing various pharmacological effects, including antioxidant, anti-inflammatory^17,18^, anti-Alzheimer’s^19,20^, anti-bacterial^21^, and anti-cancer^22–24^ activity. Recently, various studies have indicated 1,2,3-triazoles as potent anti-α-glucosidase agents^13,25–27^, such as compound **A** (Fig. 1) which is a potent competitive inhibitor of α-glucosidase. These derivatives exhibited IC_50_ values in the range of 13.0–75.5 µM^28^. A series of benzimidazole-1,2,3-triazoles containing phenoxy linker (Compound **B**) were rationally designed as α-glucosidase inhibitors and exhibited significant inhibitory activity compared with its parental compounds as well as the positive control, acarbose^29^. Recently, a series of indolinone substituted phenoxy-methyltriazole derivatives were synthesized and the derivative **C**, the most potent derivative, inhibited the enzyme competitively. In silico assessments confirmed that the phenoxy-1,2,3-triazole moiety makes compounds stable through H-bonding and pi-alkyl interactions^30^.

On the other hand, substituted acetohydrazide derivatives were introduced as the key skeleton anchoring agent that functionality interacts with amino acid residues toward the α-glucosidase. Through systematic chemical optimization, compound **D** demonstrated improved inhibition (IC_50_ = 21.9 µg/ml) against α-glucosidase compared with acarbose (IC_50_ = 34.5 µg/ml)^31^. Moreover, the moiety is perfectly nestled in the pocket of α-glucosidase^32^. Also, atranorin, a secondary metabolite of lichen, was conjugated with different hydrazines. Among the synthesized derivatives, compound **E** afforded high anti-α-glucosidase activity (IC_50_ = 6.67 µM) without toxicity on the HEK293 cell line. Recently, cyanoacetohydrazide linked to 1,2,3-triazole derivatives were found to be potent α-glucosidase inhibitors, and among them, compound **F** showed very good activity (IC_50_ = 1.00 µM) as compared with acarbose (IC_50_ = 754.1 μM). Also, fluorescence measurements confirmed conformational changes of the enzyme after binding of compound **F**^33^ (Fig. 1).

In this work, considering the structural characteristics of substituted acetohydrazide as α-glucosidase inhibitor and the advantages of 1,2,3-triazoles, a new series of *N'*-substituted benzylidene benzohydrazides linked to 1,2,3-triazoles were synthesized to evaluate their α-glucosidase inhibitory activity. Subsequently, structure−activity relationship studies (SARs) and kinetic studies were performed. Cavity detection was further executed to find a suitable site for noncompetitive inhibitors for in silico assessments.

## Results and discussion

### Chemistry

**Figure 2:**
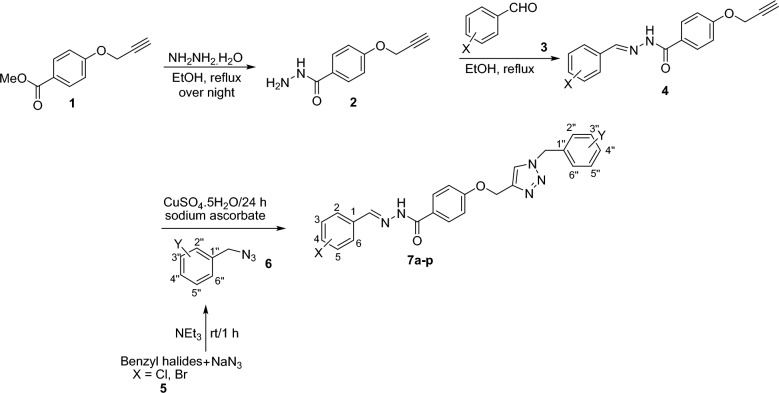
Synthesis of compounds **7a-p**.

The synthetic route for the preparation of compounds **7a-p** was schematically described in Fig. 2. It was initiated by the reaction of methyl 4-(prop-2-yn-1-yloxy)benzoate **1** and hydrazine hydrate in refluxing ethanol leading to the formation of 4-(prop-2-yn-1-yloxy)benzohydrazide **2**. Compound** 1** in turn was prepared from the reaction of 4-hydroxy benzoic acid and propargyl bromide in DMF at 80 °C. Compound **2** reacted with different aromatic aldehydes **3** in ethanol, heated at reflux catalyzed by acetic acid to afford the corresponding Schiff base derivatives** 4**. Finally, the click reaction of compound **4** and in situ prepared azides **6** in the presence of triethylamine (NEt_3_), CuSO_4_.5H_2_O, and sodium ascorbate in H_2_O/*tert*-BuOH for 24−48 h at room temperature, gave the desired products **7**.

### In vitro inhibition of α-glucosidase and the structure-activity relationships

Compounds **7a-p** were evaluated for their in vitro potential toward α-glucosidase compared with acarbose as the positive reference, and the IC_50_ values are presented in Table 1. Results indicated that all derivatives are potent α-glucosidase inhibitors compared to acarbose. Amongst, compound **7h** showed promising activity with an IC_50_ value of 0.01 µM regarded as the most potent inhibitor compared to acarbose as a positive control (IC_50_ = 752.10 μM).

**Table 1:** α-Glucosidase inhibitory activity of compounds **7a-p**.


Entry	Compound	X	Y	IC_50_ (μM)
1	**7a**	H	H	0.02 ± 0.00
2	**7b**	H	4-F	43.10 ± 0.20
3	**7c**	H	2-Cl	61.70 ± 0.30
4	**7d**	H	4-Me	0.04 ± 0.00
5	**7e**	4-NO_2_	H	3.60 ± 0.50
6	**7f.**	4-NO_2_	4-F	8.90 ± 0.10
7	**7g**	4-NO_2_	2-Cl	0.06 ± 0.00
8	**7h**	4-NO_2_	4-Me	0.01 ± 0.00
9	**7i**	4-Cl	H	261.40 ± 1.80
10	**7j**	4-Cl	4-F	648.90 ± 1.90
11	**7k**	4-Cl	2-Cl	492.47 ± 2.80
12	**7l**	4-Cl	4-Me	289.20 ± 0.30
13	**7m**	4-OMe	H	0.86 ± 0.01
14	**7n**	4-OMe	4-F	23.70 ± 0.10
15	**7o**	4-OMe	2-Cl	23.30 ± 0.70
16	**7p**	4-OMe	4-Me	101.80 ± 0.40
Acarbose				752.10 ± 1.10

A distinct inhibitory pattern was observed regarding the type of substituent at Y position on the benzyl moiety connected to the 1,2,3-triazole ring. In detail, derivatives **7a-d** (X = H) showed IC_50_ values in the range of 0.02 to 61.70 μM. Noteworthy, the presence of 4-NO_2_ substituent at X position significantly improved the inhibition and recorded IC_50_ values in the range of 0.01 to 8.09 μM. A strong electron-withdrawing group at the para position seems to have improved the activity.

Assessments on the **7i-l** bearing *para*-chlorine as halogen group at X position disclosed a considerable reduction in the potency (IC_50_ = 261.40 to 648.90 μM). However, the methoxy group at the *para* position (**7m-p**) increased the inhibitory trend with IC_50_ = 0.86 to 101.80 μM. The more detailed analysis demonstrated that the presence of NO_2_ substituent as a strong electron-withdrawing group at X (**7e-h**), improved the inhibitory potency highlighting the pivotal role of the nitro functional group for inhibition. The other potent entry was unsubstituted derivatives at X (**7a-d**) followed by 4-OMe (**7m-p**).

Precise assessments on the **7a-d** (X = H) derivatives also indicated that **7a** as an unsubstituted derivative (IC_50_ = 0.02 μM) and **7d** bearing 4-methyl group at Y, as a lipophilic and weak electron-donating group (IC_50_ = 0.04 μM), were more potent in comparison to derivatives bearing 4-F and 2-Cl in this set. The evaluations on **7e-h** (X = NO_2_) as the most potent set of derivatives showed the flowing order of potency 4-Me (**7h**) ˃ 2-Cl (**7g**) ˃ H (**7e**) ˃ 4-F (**7f**). As can be seen in these two sets of derivatives, 4-Me substituted analog at Y exhibited good potency. A different trend was observed in the **7i-l** bearing 4-Cl and **7m-p** containing 4-OMe at X, so that **7l** (X = 4-Cl, Y = 4-Me) and **7p** (X = 4-OMe, X = 4-Me) were not as potent as their counterparts. In the two mentioned sets of compounds, the unsubstituted derivatives in each set, compounds **7i** (X = 4-Cl, Y = H, IC_50_ = 261.40 μM) and **7m** (X = 4-OMe, Y = H, IC_50_ = 0.86 μM) were highly potent.

To sum up, the straightforward SAR was extracted so that the presence of 4-NO_2_ as a bulk-electron withdrawing group at X, regardless of the type of substituent at Y significantly improved the inhibitory activity of compounds against α-glucosidase. Based on Hammett electronic σ and Hansch π constants, the electronic effect and lipophilicity of the substituents can affect the biological activity^34^. Herein, the α-glucosidase inhibitory activities of compounds **7e**-**h** were influenced by the NO_2_ group (σ = 0.78, π = − 0.28), and generally, high activity was obtained by these compounds. However, the right-forward trend cannot be seen in Hammett's electronic properties, so compounds **7i-l** (X = Cl, σ = 0.23, π = 0.71) exhibited lower activity in comparison with compounds **7m-p** (X = OMe, σ = -− 0.27, π = − 0.02)^35^ which were supposed to be weak inhibitors due to low σ value. It seems that the effects of Y substituents should not be ignored. Also, it was revealed that the increased lipophilicity at the X position reduced the potency as high lipophilic Cl-derivatives **7i**-**l** gave lower activity against α-glucosidase.

Unsubstituted compounds at the X and Y positions can also be categorized as the second top potent group. Investigation on Y showed different patterns so that if X = 4-Cl or OMe, the unsubstituted derivative at Y is more favorable, and the Me group is inferior to that in the potency. Vice versa, 4-Me moiety seems to improve the inhibitory activity in the X = NO_2_ set of compounds.

The SAR comparison between the designed compounds and previously reported scaffolds was executed. The benzimidazole-1,2,3-triazole hybrids demonstrated IC_50_ values ranging from 25.2 to 176.5 μM. SARs exhibited that the presence of ethyl or methyl as lipophilic electron-donating substituent on the benzyl ring improved the potency *vs* halogen and electron-withdrawing groups. The exception returned to 2-NO_2_ moiety, which was categorized as the second top potent derivative^36^.

Hydrazineylideneindolinone linked to phenoxymethyl-1,2,3-triazole derivatives showed IC_50_ in the range of 16.43 to > 750 µM. Interesting results were obtained with SAR analysis, and it was concluded that the presence of a substituent on the phenoxy linker deteriorated the potency, and fluoride substituent on the terminal benzyl ring induced better α-glucosidase inhibitory activity compared to other derivatives^30^. Recently, novel series of cyanoacetohydrazide linked to phenoxy-triazole was developed (IC_50_ = 1.00 to 750 against α-glucosidase), and different results were obtained in this case. The presence of methoxy on the phenoxy linker was favorable, and fluorine and chlorine substituents on the benzyl ring empowered the activity^37^. Although the backbones of these derivatives are not similar to fully extracting the SARs; it was understood that nitro group at the X position is favorable and in this case, 4-Me moiety at Y seems to increase the activity (Supplementary Information 1).

### Enzyme kinetic studies

**Figure 3:**
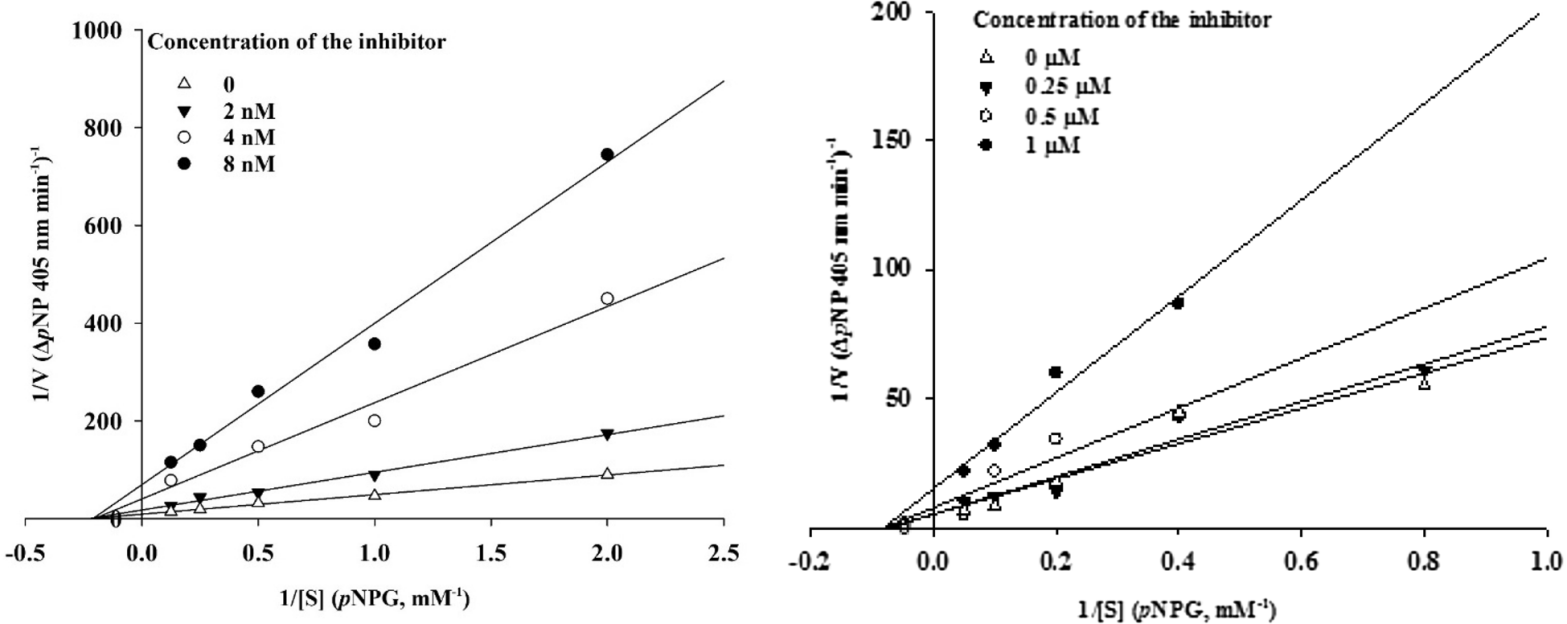
Kinetic study of α-glucosidase inhibition by compounds **7h** (left) and **7a** (right).

A kinetic study was achieved to investigate the mode of inhibition of α-glucosidase by compounds **7a** and **7h**. According to Fig. 3, the Lineweaver−Burk plots showed that the *K*_m_ gradually increased, and *V*_*max*_ remained unchanged with increasing the inhibitors concentration, indicating a noncompetitive inhibitory activity, with *K*_i_ = 0.01 and 0.02 μM for compounds **7h** and **7a**, respectively.

### Fluorescence spectroscopy

**Figure 4:**
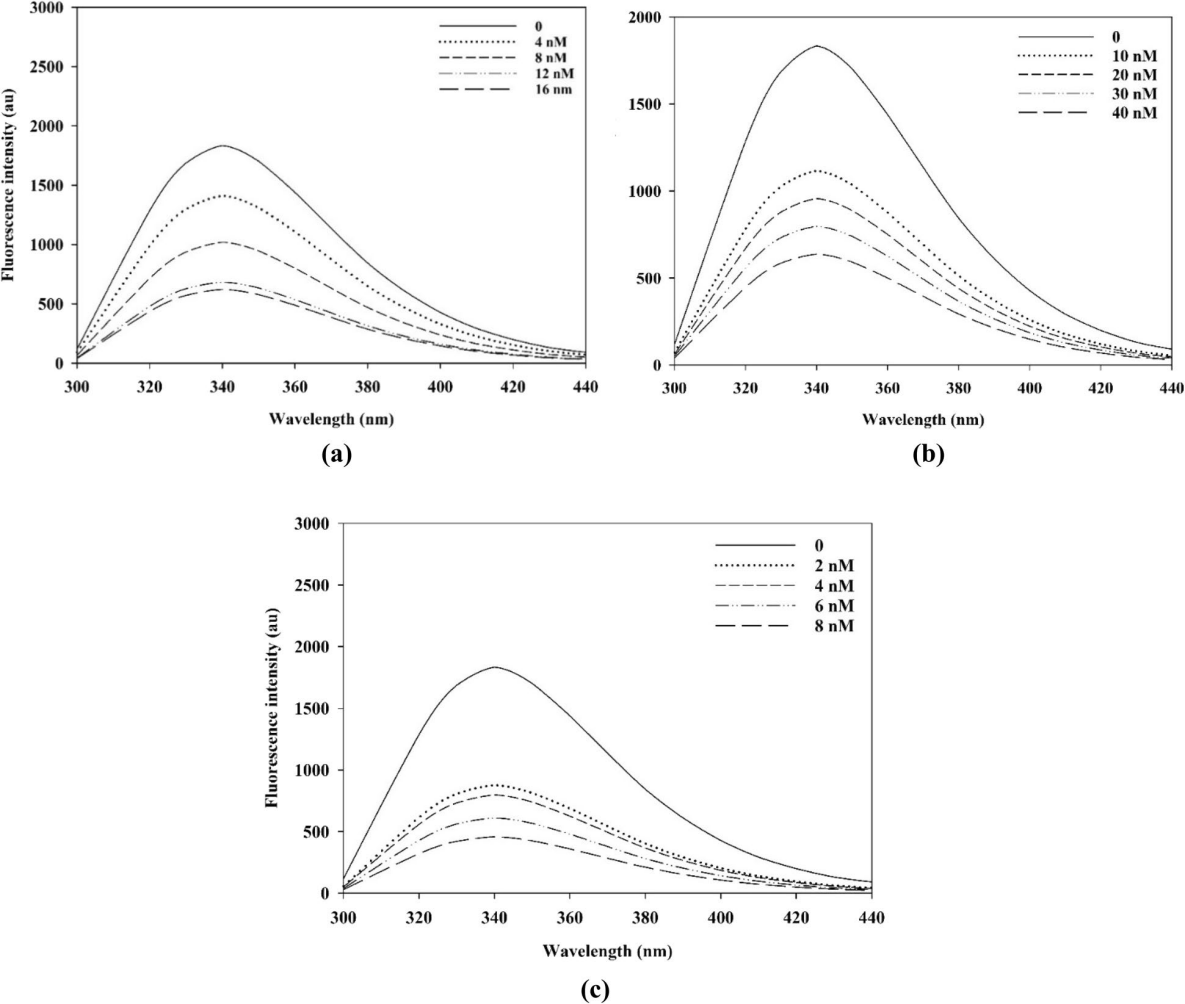
Fluorescence spectra of α-glucosidase in the presence of compounds **7a** (**a**), **7d** (**b**) and **7h **(**c**) at 25 °C.

Fluorescence spectrometry was used for studying the interactions between selected α-glucosidase inhibitors (**7a**, **7d**, and **7h**) and the enzyme at 25 °C and pH 6.8 to determine the binding constants, number of binding sites, and thermodynamic parameters of the corresponding interactions. Herein, the maximum fluorescence intensity of α-glucosidase was obtained at 340 nm, which can be attributed to tryptophan, tyrosine, and phenylalanine residues that can act as intrinsic fluorescence probes with excitation at 280 nm. As shown in Fig. 4, the fluorescence intensity of α-glucosidase was gradually decreased in the presence of compounds **7a**, **7d**, and **7h** in a concentration dependent manner at 340 nm, indicating that those compounds interacted with α-glucosidase and may change the fluorescence characteristics of the enzyme.

The fluorescence quenching process was characterized by the Stern−Volmer equation (experimental section, Eq. 1). As shown in Table 2, the quenching model was static as the quenching rate constant values *K*_q_ (L mol^−1^ s^−1^) for selected inhibitors **7a**, **7d**, and **7h** at 25 and 35 ºC were obtained much higher than 2.0 × 10^10^ L mol^−1^ s^−1^ which is the maximum collision rate constant for all quencher compounds that collide with biological macromolecules.

**Table 2:** Calculated quenching rate constants for inhibitors **7a**, **7d**, and **7h** at various temperatures.

Compound	*K*_q_ (L mol^−1^ s^−1^)	
	25 °C	35 °C
**7a**	1.5 × 10^15^	9.0 × 10^15^
**7d**	4.2 × 10^15^	1.0 × 10^15^
**7h**	1.0 × 10^15^	6.8 × 10^15^

In the static quenching, it is assumed that there are the same and independent binding sites (*n*) in the protein (herein α-glucosidase) which all are potent to construct interactions with the inhibitor (experimental section, Eq. 2).

**Figure 5:**
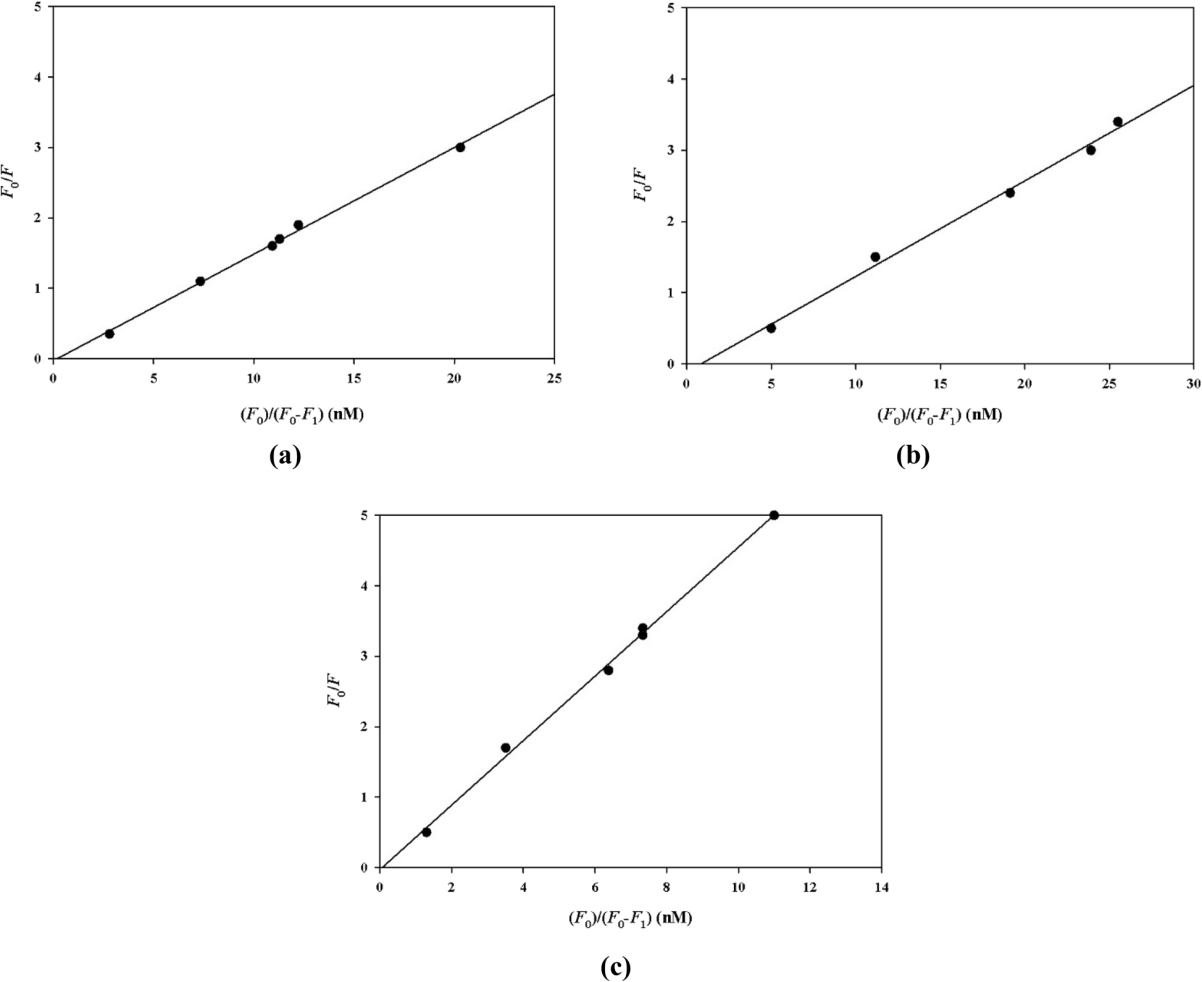
The plots* F*_0_/*F* vs function of [*D*_t_] *F*_0_/(*F*_0_ − *F*) at 25 °C for **7a** (**a**), **7d** (**b**)  and **7h** (**c**).

Plots of fluorescence intensity ratio (*F*_0_/*F*) versus [*D*_t_] *F*_0_/(*F*_0_ − *F*) were obtained (experimental section, Eqs. 3–5) by keeping the total concentrations of α-glucosidase at 0.1 µM and varying the total concentrations of compounds **7a**, **7d**, and **7h** (Fig. 5) to calculate the binding constants and binding sites (*n*) at different temperatures (Table 3). Among investigated inhibitors, compound **7d** demonstrated the lowest binding constant and the highest value was calculated for compound **7h**.

**Table 3:** Binding constants and binding sites for inhibitors **7a**, **7d**, **7h** at different temperatures.

	25 °C			35 °C
Compound	*K*_A_ (L mol^−1^ s^−1^)	*n*	*r*^*a*^	*K*_A_ (L mol^−1^ s^−1^)
**7a**	15.1 × 10^7^	0.8	0.999	7.0 × 10^7^
**7d**	13.4 × 10^7^	0.1	0.997	5.0 × 10^7^
**7h**	45.8 × 10^7^	0.06	0.999	23.3 × 10^7^

^a^r is the regression coefficient.

Finally, the acting forces between selected inhibitors (**7a**, **7d**, and **7h**) and α-glucosidase were determined (Table 4) according to the standard thermodynamic values (experimental section, Eqs. 6 and 7). These forces may include Van der Waals, electrostatic, hydrogen bonding, and hydrophobic interactions. The Δ*H* values for three inhibitors were less than zero (Δ*H* < 0) and those of Δ*S* were all greater than 0 (Δ*S* > 0). These results indicated that the acting force between these inhibitors and α-glucosidase was mainly electrostatic interactions.

**Table 4:** Standard thermodynamic values.

Compound	Δ*G* (kJ mol^−1^)	Δ*H* (kJ mol^−1^)	Δ*S* (J mol^−1^)
**7a**	− 45.23	− 31.25	48.37
**7d**	− 46.28	− 4.07	146.0
**7h**	− 49.27	− 28.08	71.1

### Molecular docking studies

To achieve more information on the molecular mechanisms behind the ability to inhibit the α-glucosidase, designed compounds were docked into the target enzyme. First, to validate docking assessments, self-docking was performed with an actual inhibitor obtained from the PDB (5NN8). The top-ranked docking score of the crystallographic inhibitor was superimposed over the X-ray coordinate of the experimentally derived structure and showed an RMSD of 1.9 Å (RMSD < 2 Å is the acceptable value).

**Figure 6:**
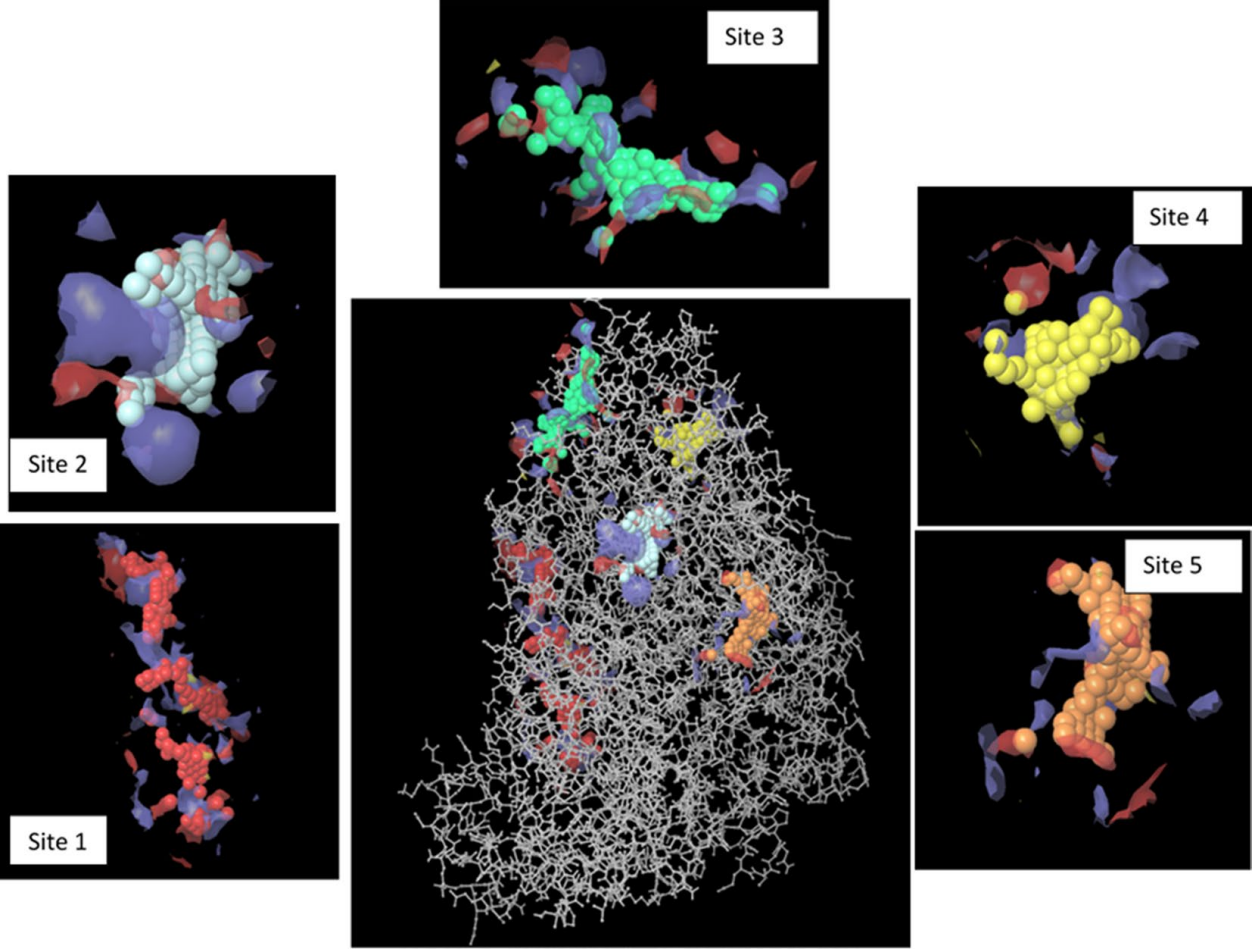
Potential binding sites of the α-glucosidase detected by cavity detection tools.

The enzyme kinetic study showed that compounds **7a** and **7h** acted as noncompetitive inhibitors. In this type of inhibition, the inhibitor binds to the enzyme at a location other than the active site. To find the suitable sites, the enzyme was subjected to mastreo sitemap tool to find the possible cavity of the enzyme. Five possible binding sites were detected on the surface of the enzyme (Fig. 6) which can be suitable for noncompetitive inhibition. Next, compound **7h** was docked in all the potential binding sites of the enzyme as the most potent structure.

**Figure 7:**
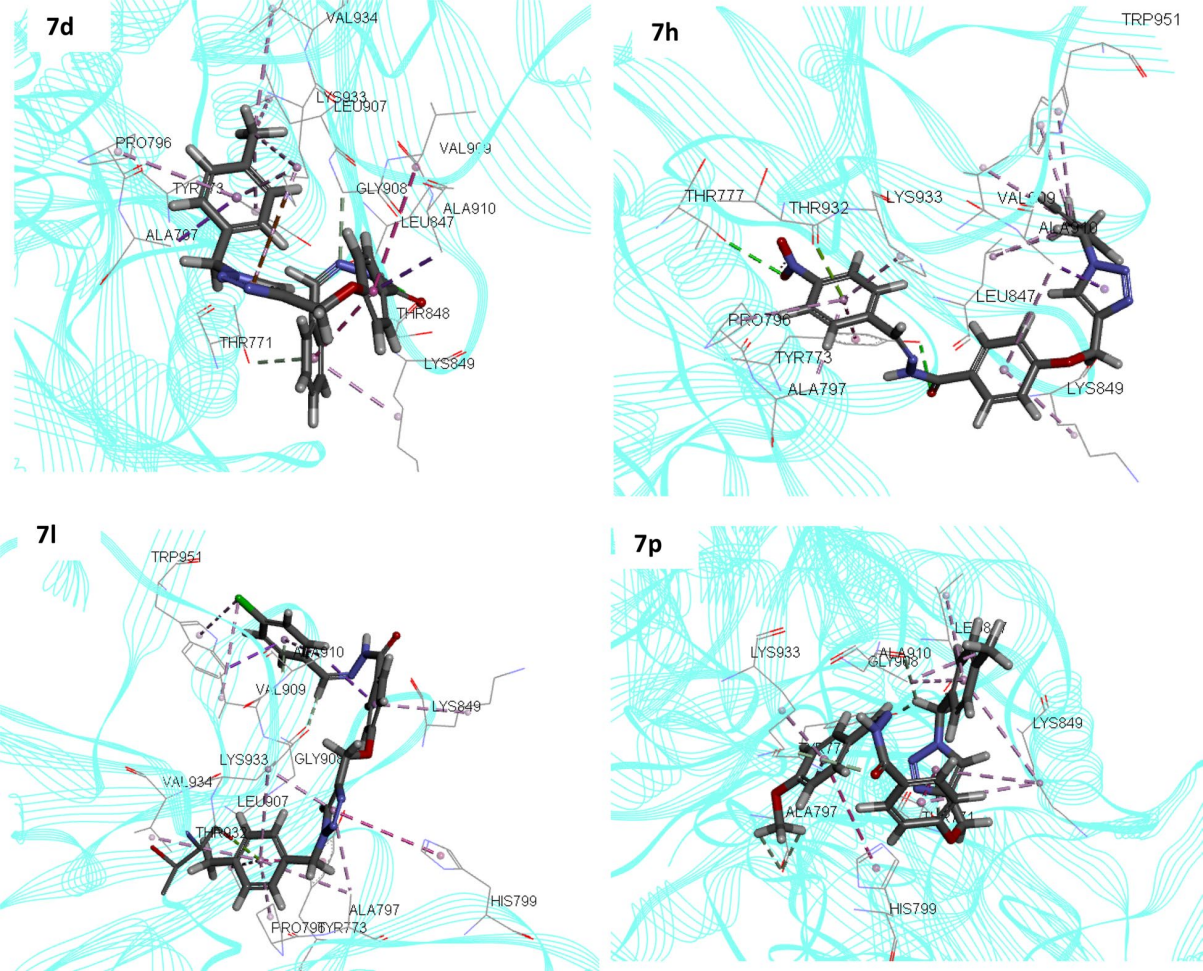
The proposed binding modes of compounds **7d**, **7h**, **7l**, and **7p** with the allosteric site of α-glucosidase.

Considering the Moledock score and interactions, site 4 appeared to have the maximum affinity in comparison to other identified sites. Next, the same docking procedure was applied to all selected derivatives at site 4. In this respect, derivatives **7h** and **7d** as the potent inhibitors and **7l** as the least active analog as well as **7p** were docked within the allosteric site (Y = methyl in all selected compounds). Residues involved in the interaction of these compounds plus MolDock Score were shown in Table 5 and 3D structures of each ligand were presented in Fig. 7.

**Table 5:** The predicted binding affinity of the selected ligands with α-glucosidase.

Compound	MolDock score (KJ/mol)	Interactions	Residues
**7d**	− 120.57	Hydrogen Bonding	Leu847
		Hydrogen Bonding	Thr848
		Pi-Donor Hydrogen Bonding	Thr771
		Pi-Donor Hydrogen Bonding	Gly908
		Pi-Sigma	Ala797
		Pi-Sigma	Ala910
		Pi-Cation	Lys933
		Pi-Alkyl	Lys849
		Pi-Alkyl	Lys933
		Pi-Alkyl	Pro796
		Alkyl	Lys933
		Alkyl	Pro796
		Alkyl	Leu907
		Alkyl	Val934
**7h**	− 148.49	Hydrogen Bonding	Tyr773
		Hydrogen Bonding	Thr777
		Hydrogen Bonding	Thr932
		Carbon Hydrogen Bonding	Lys849
		Pi-Lone pair	Thr932
		Pi-Sigma	Ala910
		Pi-Alkyl	Pro796
		Pi-Alkyl	Ala797
		Pi-Alkyl	Lys933
		Pi-Alkyl	Lys849
		Pi-Alkyl	Val909
		Pi-Alkyl	Ala910
		Pi-Alkyl	Leu847
		Alkyl	Leu847
		Alkyl	Trp951
**7l**	− 89.89	Carbon Hydrogen Bonding	Gly908
		Pi-Donor Hydrogen Bonding	Ala910
		Pi-Sigma	Val909
		Pi-Sigma	Ala910
		Pi-Pi T-shaped	Tyr773
		Pi-Alkyl	Ala797
		Pi-Alkyl	Pro796
		Pi-Alkyl	Leu907
		Pi-Alkyl	Val934
		Pi-Alkyl	Lys849
		Pi-Alkyl	Lys933
		Alkyl	Trp951
		Unfavorable	Ala797
		Unfavorable	Pro796
		Unfavorable	Leu907
		Unfavorable	Val934
**7p**	− 102.09	Carbon Hydrogen Bonding	Thr771
		Carbon Hydrogen Bonding	Leu847
		Carbon Hydrogen Bonding	Thr773
		Carbon Hydrogen Bonding	Gly908
		Carbon Hydrogen Bonding	Ala797
		Carbon Hydrogen Bonding	Lys933
		Pi-Donor Hydrogen Bonding	Thr771
		Pi-Lone Pair	Gly908
		Pi-Pi T-shaped	His799
		Pi-Alkyl	Lys933
		Pi-Alkyl	Ala797
		Pi-Alkyl	Lys849
		Pi-Alkyl	Ala910
		Alkyl	Ala910

At first look, it can be seen that the order of IC_50_ values was consistent within the MolDock Score values so that compounds **7h** and **7d** as potent inhibitors in the list, IC_50_ = 0.01 and 0.04 µM, generated the MolDock Score value of − 148.49 and − 120.57 kJ/mol. Compound **7l** with IC_50_ value of 289.20 µM generated a low score value of − 89.89 kJ/mol which showed that the in silico assessments could successfully categorize compounds based on their experimental activities. Also, the least potent compounds recorded some unfavorable interactions and were unable to participate in strong interactions with the proposed site while **7h** and **7d** effectively participate in H-bonding interactions with the allosteric site.

### Physicochemical properties

According to the physicochemical properties predicted from SwissADME web tool, all compounds exhibited desirable molecular properties with no drug-likeness rules violations (Table 6). All inhibitors also showed a desirable bioavailability score.

**Table 6:** Physicochemical properties of synthesized derivatives.

	Physicochemical Properties							Drug-likeness rules					
Compounds	MW (g/mol)	Num. rotatable bonds	Num. H-bond acceptors	Num. H-bond donors	Log P o/w	Molar Refractivity	TPSA (Å^2^)	Lipinski (Pfizer)	Ghose (Amgen)	Veber (GSK)	Egane (Pharmacia)	Mugge (Bayer)	Abbott bioavailability score
**7a**	411.46	9	5	1	3.36	117.92	81.40	Yes	Yes	Yes	Yes	Yes	0.55
**7b**	429.45	9	6	1	3.79	117.88	81.40	Yes	Yes	Yes	Yes	Yes	0.55
**7c**	445.90	9	5	1	4.32	122.93	81.40	Yes	Yes	Yes	Yes	Yes	0.55
**7d**	425.48	9	5	1	2.99	122.88	81.40	Yes	Yes	Yes	Yes	Yes	0.55
**7e**	457.46	10	7	2	3.06	122.99	121.71	Yes	Yes	Yes	Yes	Yes	0.55
**7f**	475.45	10	8	2	3.16	122.95	121.71	Yes	Yes	Yes	Yes	Yes	0.55
**7g**	491.91	10	7	2	3.69	128.00	121.71	Yes	Yes	Yes	Yes	Yes	0.55
**7h**	471.49	10	7	2	3.02	127.96	121.71	Yes	Yes	Yes	Yes	Yes	0.55
**7i**	445.90	9	5	1	4.32	122.93	81.40	Yes	Yes	Yes	Yes	Yes	0.55
**7j**	463.89	9	6	1	4.42	122.89	81.40	Yes	Yes	Yes	Yes	Yes	0.55
**7k**	480.35	9	5	1	4.95	127.94	81.40	Yes	Yes	Yes	Yes	Yes	0.55
**7l**	459.93	9	5	1	4.69	127.89	81.40	Yes	Yes	Yes	Yes	Yes	0.55
**7m**	441.48	10	6	1	3.67	124.41	90.63	Yes	Yes	Yes	Yes	Yes	0.55
**7n**	459.47	10	7	1	3.77	124.37	90.63	Yes	Yes	Yes	Yes	Yes	0.55
**7o**	475.93	10	6	1	4.29	129.42	90.63	Yes	Yes	Yes	Yes	Yes	0.55
**7p**	455.51	10	6	1	3.85	129.38	90.63	129.38	129.38	129.38	129.38	129.38	129.38

In the case of molar refractivity values which were related to the overall bulkiness and lipophilicity of the synthesized compounds, the most potent compound **7h,** depicted a relatively high value (127.96). However, compound **7a** showed a low value of 117.92, indicating no definite relation between the bulkiness and α-glucosidase inhibition, which can be confirmed in the series of compounds **7a**−**p**.

## Conclusion

Following our expertise in the rational design of α-glucosidase inhibitors; herein, a series of *N'*-substituted benzylidene benzohydrazides linked to 1,2,3-triazoles were designed and synthesized.

The chemical structures of all derivatives were characterized using NMR, FTIR, MS spectrometry, and elemental analysis. All compounds exhibited pronounced anti-α-glucosidase activity with IC_50_ values in the range of 0.01−648.90 μM compared with the reference compound, acarbose (IC_50_ = 752.10 µM). The SAR data showed that the designed backbone was highly potent against α-glucosidase and the incorporation of a *para*-nitro moiety on the benzyl moiety connected to 1,2,3-triazole ring significantly improved the potency. Compounds **7a** (IC_50_ = 0.02 μM) and **7h** (IC_50_ = 0.01 μM) as the most potent inhibitors, were subjected to kinetic experiments and revealed the noncompetitive inhibition pattern. In addition, fluorescence spectroscopy demonstrated that the intrinsic fluorescence of α-glucosidase was quenched by three inhibitors **7a**, **7d**, and **7h,** due to the formation of inhibitor-fluorophore complex, resulting in the decrease in enzyme activity. This result was in good agreement with our results obtained from the kinetic studies. It should be noted that the calculation of standard thermodynamic values (ΔG, ΔH, and ΔS) revealed that the acting force between these inhibitors and the α-glucosidase was mainly electrostatic interactions. In the docking study, mastreo sitemap tools were applied to find the allosteric site for noncompetitive inhibitors and an appropriate cavity was proposed. To evaluate the behavior of derivatives within the proposed site, enzyme molecular docking assessments were performed. Derivatives **7d** and **7h** showed the lowest score and could effectively participate in H-bonding interactions with the proposed site, while derivatives with fewer activities exhibited no strong interactions with the allosteric site. This study has made good progress in anti-T2DM agents and promoted the development of related fields.

## Method and materials

Melting points were measured on a Electrothermal digital melting point apparatus IA9300 and are uncorrected. ^1^H- and ^13^C-NMR spectra were recorded on a Varian-INOVA 500 MHz, using TMS as an internal standard. IR spectra were obtained on a Nicolet Magna FTIR 550 spectrophotometer (KBr disks). Mass spectra were recorded on an Agilent Technology (HP) mass spectrometer operating at an ionization potential of 70 eV. Elemental analysis was performed on an Elementar Analysensystem GmbH VarioEL CHNS mode.

### Synthesis of compounds 7

A mixture of methyl 4-(prop-2-yn-1-yloxy)benzoate **1** (1 mmol) and hydrazine hydrate (4 mmol) in EtOH (10 ml) was heated at reflux overnight. After completion of the reaction, the mixture was poured over ice and water, and the precipitate was filtered off to give 4-(prop-2-yn-1-yloxy)benzohydrazide **2**. Next, a mixture of compound **2** (1 mmol) and aldehyde **3** (1 mmol) in EtOH (15 ml), in the presence of a few drops acetic acid was heated at reflux for 24 h. After completion of the reaction, the mixture was poured over ice and water and the precipitate was filtered off to afford compound **4**. Finally, the click reaction was conducted by a mixture of compound **4** and in situ prepared azide derivative **6**^30^. For this purpose, benzyl chloride/bromide derivative **5** (1.1 mmol) and sodium azide (0.06 g, 0.9 mmol) in the presence of triethylamine (0.13 g, 1.3 mmol) in water (4 ml) and *tert*-butyl alcohol (4 ml) was stirred at room temperature for 30 min. Next, compound **4** (0.5 mmol) and CuSO_4_·5H_2_O (7 mol%) were added to the reaction mixture and it was continued for 24−48 h at the same temperature. After completion of the reaction as checked by TLC, the mixture was poured on the crushed ice, the precipitates were filtered off and washed with water. All products were recrystallized from ethyl acetate and petroleum ether.

#### 4-((1-Benzyl-1H-1,2,3-triazol-4-yl)methoxy)-*N*′-benzylidenebenzohydrazide (7a)

Gray precipitates, Yield: 78%, mp 248−251 °C. IR (KBr, cm^−1^): 3417, 3051, 2924, 2850, 1687, 1635, 1589. ^1^HNMR (500 MHz, DMSO-*d*_*6*_): 11.75 (s, 1H, NH), 8.50 (s, 1H, CH), 8.29 (s, 1H, triazole), 7.91 (d,* J* = 8.3 Hz, 2H, H3′, H5′), 7.78 (d, *J* = 7.0 Hz, 2H, H2, H6), 7.50−7.46 (m, 3H, H3, H4, H5), 7.35−7.19 (m, 7H, H2′, H6′, H2′′, H3′′, H4′′, H5′′, H6′′), 5.62 (s, 2H, CH_2_), 5.21 (s, 2H, CH_2_) ppm. ^13^CNMR (125 MHz, DMSO-*d*_*6*_): 163.1, 161.3, 147.5, 142.6, 133.6, 133.2, 131.1, 129.7, 129.1, 128.9, 128.3, 127.5, 125.4, 125.1, 122.8, 114.5, 61.5, 52.3 ppm. MS (m/z, %): 411 (M^+^, 3), 295 (8), 245 (27), 182 (33), 154 (51), 121 (23), 105 (87), 77 (55), 57 (100). Calcd for C_24_H_21_N_5_O_2_: C, 70.06; H, 5.14; N, 17.02. Found: C, 70.23; H, 5.05; N, 16.91.

#### *N*′-Benzylidene-4-((1-(4-fluorobenzyl)-1H-1,2,3-triazol-4-yl)methoxy)benzohydrazide (7b)

Gray precipitates, Yield: 78%, mp 160−163 °C. IR (KBr, cm^−1^): 3415, 3051, 2922, 2851, 1687, 1636, 1590. ^1^HNMR (500 MHz, DMSO-*d*_*6*_): 11.73 (s, 1H, NH), 8.45 (s, 1H, CH), 8.31(s, 1H, triazole), 7.89 (d, *J* = 8.5 Hz, 2H, H3′, H5′), 7.71 (d, *J* = 7.0 Hz, 2H, H2, H6), 7.45−7.38 (m, 5H, H3, H4, H5, H2′′, H6′′), 7.20−7.14 (m, 4H, H2′, H6′, H3′′, H5′′), 5.60 (s, 2H, CH_2_), 5.22 (s, 2H, CH_2_) ppm. ^13^CNMR (125 MHz, DMSO-*d*_*6*_): 163.3, 162.4, 161.3, 147.7, 146.4, 143.1, 134.7, 132.6, 130.8, 129.9, 129.3, 128.6, 127.4, 125.2, 116.0, 114.9, 61.7, 52.5 ppm. Calcd for C_24_H_20_FN_5_O_2_: C, 67.12; H, 4.69; N, 16.31. Found: C, 66.98; H, 4.53; N, 16.59.

#### *N*′-Benzylidene-4-((1-(2-chlorobenzyl)-1H-1,2,3-triazol-4-yl)methoxy)benzohydrazide (7c)

White precipitates, Yield: 80%, mp 203−207 °C. IR (KBr, cm^−1^): 3416, 3052, 2927, 2848, 1688, 1631, 1588. ^1^HNMR (500 MHz, DMSO-*d*_*6*_): 11.72 (s, 1H, NH), 8.44 (s, 1H, CH), 8.28 (s, 1H, triazole), 7.90 (d,* J* = 8.3 Hz, 2H, H3′, H5′), 7.71 (d, *J* = 7.0 Hz, 2H, H2, H6), 7.52 (d*, J* = 7.5 Hz, 1H, H3′′), 7.45−7.35 (m, 5H, H3, H4, H5, H4′′, H5′′), 7.23 (d, *J* = 7.5 Hz, 1H, H6′′), 7.16 (d, *J* = 8.3 Hz, 2H, H2′, H6′), 5.72 (s, 2H, CH_2_), 5.24 (s, 2H, CH_2_) ppm. ^13^CNMR (125 MHz, DMSO-*d*_*6*_): 163.1, 162.6, 147.6, 142.9, 137.8, 134.9, 133.6, 133.1, 131.0, 130.7, 130.4, 130.1, 129.9, 128.7, 128.2, 127.4, 125.1, 114.9, 61.6, 51.1 ppm. Calcd for C_24_H_20_ClN_5_O_2_: C, 64.65; H, 4.52; N, 15.71. Found: C, 65.02; H, 4.41; N, 15.45.

#### *N*′-Benzylidene-4-((1-(4-methylbenzyl)-1H-1,2,3-triazol-4-yl)methoxy)benzohydrazide (7d)

Gray precipitates, Yield: 75%, mp 154−156 °C. IR (KBr, cm^−1^): 3417, 3050, 2924, 2853, 1690, 1637, 1587. ^1^HNMR (500 MHz, DMSO-*d*_*6*_): 11.76 (s, 1H, NH), 8.47 (s, 1H, CH), 8.29 (s, 1H, triazole), 7.90−7.72 (m, 4H, H2, H6, H3′, H5′), 7.51−7.47 (m, 3H, H3, H4, H5), 7.22−7.14 (m, 6H, H2′, H6′, H2′′, H3′′, H5′′, H6′′), 5.55 (s, 2H, CH_2_), 5.19 (s, 2H, CH_2_), 2.27 (s, 3H, CH_3_) ppm. ^13^CNMR (125 MHz, DMSO-*d*_*6*_): 163.1, 161.4, 146.1, 143.8, 138.0, 135.1, 134.8, 133.4, 132.00, 129.7, 129.4, 129.4, 129.3, 128.5, 127.5, 114.9, 60.8, 53.4, 21.5 ppm. Calcd for C_25_H_23_N_5_O_2_: C, 70.57; H, 5.45; N, 16.46. Found: C, 70.88; H, 5.22; N, 16.13.

#### 4-((1-Benzyl-1H-1,2,3-triazol-4-yl)methoxy)-*N*′-(4-nitrobenzylidene)benzohydrazide (7e)

Yellow precipitates, yield: 48%, mp 193−196 °C. IR (KBr, cm^−1^): 3415, 3052, 2926, 2851, 1687, 1630, 1586, 1533, 1375. ^1^HNMR (500 MHz, DMSO-*d*_*6*_): 12.04 (s, 1H, NH), 8.54 (s, 1H, CH), 8.33 (s, 1H, triazole), 8.30−7.97 (m, 6H, H2, H3, H5, H6, H3′, H5′), 7.35−7.22 (m, 5H, H2′′, H3′′, H4′′, H5′′, H6′′), 7.18 (d, *J* = 8.3 Hz, 2H, H2′, H6′), 5.62 (s, 2H, CH_2_), 5.24 (s, 2H, CH_2_). ^13^CNMR (125 MHz, DMSO-*d*_*6*_): 162.0, 160.1, 147.7, 144.6, 140.8, 136.0, 129.7, 128.8, 128.2, 128.0, 127.9, 125.1, 124.9, 124.1, 121.0, 114.5, 61.3, 52.9 ppm. Calcd for C_24_H_20_N_6_O_4_: C, 63.15; H, 4.42; N, 18.41. Found: C, 63.29; H, 4.73; N, 18.57.

#### 4-((1-(4-Fluorobenzyl)-1H-1,2,3-triazol-4-yl)methoxy)-*N*′-(4-nitrobenzylidene)benzohydrazide (7f)

Light green precipitates, Yield: 75%, mp 202−205 °C. IR (KBr, cm^−1^): 3415, 3050, 2925, 2850, 1684, 1635, 1590, 1535, 1375. ^1^HNMR (500 MHz, DMSO-*d*_*6*_): 12.03 (s, 1H, NH), 8.53 (s, 1H, CH), 8.31−8.29 (m, 3H, H3, H5, triazole), 7.97−7.92 (m, 4H, H2, H6, H3′, H5′), 7.39−7.38 (m, 2H, H2′′, H6′′), 7.22−7.17 (m, 4H, H2′, H6′, H3′′, H5′′), 5.60 (s, 2H, CH_2_), 5.23 (s, 2H, CH_2_) ppm. ^13^CNMR (125 MHz, DMSO-*d*_*6*_): 163.3, 161.4, 160.9 (d, *J*_*C−F*_ = 256.9 Hz), 148.1, 145.1, 143.1, 141.2, 132.7, 130.8 (d, *J*_*C−F*_ = 8.1 Hz), 129.6, 128.3, 125.7, 125.2, 124.5, 116.1 (d, *J*_*C−F*_ = 21.5 Hz), 114.9, 61.7, 52.5 ppm. Calcd for C_24_H_19_FN_6_O_4_: C, 60.76; H, 4.04; N, 17.71. Found: C, 60.88; H, 3.87; N, 17.42.

#### 4-((1-(2-Chlorobenzyl)-1H-1,2,3-triazol-4-yl)methoxy)-*N*′-(4-nitrobenzylidene)benzohydrazide (7g)

Yellow precipitates, Yield: 83%, mp 204−206 °C. IR (KBr, cm^−1^): 3417, 3051, 2922, 2853, 1686, 1634, 1587, 1535, 1375.^1^HNMR (500 MHz, DMSO-*d*_*6*_): 12.03 (s, 1H, NH), 8.53 (s, 1H, CH), 8.29−8.27 (m, 3H, H3, H5, triazole), 7.96 (d, *J* = 8.2 Hz, 2H, H2, H6), 7.91 (d, *J* = 8.3 Hz, 2H, H3′, H5′) 7.51 (d, *J* = 7.5 Hz, 1H, H3′′), 7.41−7.35 (m, 2H, H4′′, H5′′), 7.23 (d, *J* = 7.5 Hz, 1H, H6′′), 7.17 (d, *J* = 8.3 Hz, 2H, H2′, H6′,), 5.72 (s, 2H, CH_2_), 5.25 (s, 2H, CH_2_) ppm. ^13^CNMR (125 MHz, DMSO-*d*_*6*_): 162.4, 161.2, 148.2, 145.1, 142.9, 141.2, 133.6, 133.1, 131.0, 130.7, 130.1, 129.3, 128.3, 128.2, 125.8, 125.7, 124.5, 115.0, 61.7, 51.1 ppm. Calcd for C_24_H_19_ClN_6_O_4_: C, 58.72; H, 3.90; N, 17.12. Found: C, 58.41; H, 4.14; N, 17.29.

#### 4-((1-(4-Methylbenzyl)-1H-1,2,3-triazol-4-yl)methoxy)-*N*′-(4-nitrobenzylidene)benzohydrazide (7h)

Brown precipitates, yield: 38%, mp 247−250 °C. IR (KBr, cm^−1^): 3416, 3052, 2925, 2851, 1690, 1634, 1585, 1536, 1372. ^1^HNMR (500 MHz, DMSO-*d*_*6*_): 12.04 (s, 1H, NH), 8.54 (s, 1H, CH), 8.29- 7.93 (m, 7H, H2, H3, H5, H6, H3′, H5′, triazole), 7.22−7.18 (m, 6H, H2′, H6′, H2′′, H3′′, H5′′, H6′′), 5.56 (s, 2H, CH_2_), 5.23 (s, 2H, CH_2_), 2.27 (s, 3H, CH_3_). ^13^CNMR (125 MHz, DMSO-*d*_*6*_): 161.8, 160.1, 147.7, 145.8, 140.8, 137.5, 132.9, 129.6, 129.2, 128.0, 127.5, 127.3, 124.7, 124.0, 120.0, 114.5, 61.2, 52.7, 20.6 ppm. MS (m/z, %): 470 (M^+^, 2), 207 (7), 182 (15), 161 (100), 105 (72), 77 (41), 57 (58). Calcd for C_25_H_22_N_6_O_4_: C, 63.82; H, 4.71; N, 17.86. Found: C, 64.17; H, 4.82; N, 17.61.

#### 4-((1-Benzyl-1H-1,2,3-triazol-4-yl)methoxy)-*N*′-(4-chlorobenzylidene)benzohydrazide (7i)

Light green precipitates, yield: 51%, mp 211−214 °C. IR (KBr, cm^−1^): 3415, 3048, 2924, 2853, 1690, 1627, 1588. ^1^HNMR (500 MHz, DMSO-*d*_*6*_): 11.72 (s, 1H, NH), 8.44 (s, 1H, CH), 8.33 (s, 1H, triazole), 7.90 (d, *J* = 8.3 Hz, 2H, H3′, H5′), 7.76−7.15 (m, 11H, H2, H3, H4, H5, H2′, H6′, H2′′, H3′′, H4′′, H5′′, H6′′), 5.62 (s, 2H, CH_2_), 5.24 (s, 2H, CH_2_). ^13^CNMR (125 MHz, DMSO-*d*_*6*_): 162.0, 160.5, 147.5, 141.5, 135.9, 134.1, 132.0, 129.5, 128.9, 128.7, 128.2, 128.0, 127.0, 124.9, 120.5, 114.4, 61.2, 52.9 ppm. Calcd for C_24_H_20_ClN_5_O_2_: C, 64.65; H, 4.52; N, 15.71. Found: C, 64.92; H, 4.46; N, 15.88.

#### *N*′-(4-Chlorobenzylidene)-4-((1-(4-fluorobenzyl)-1H-1,2,3-triazol-4-yl)methoxy)benzohydrazide (7j)

Orange precipitates, yield: 49%, mp 236−238 °C. IR (KBr, cm^−1^): 3417, 3051, 2925, 2852, 1687, 1632, 1586. ^1^HNMR (500 MHz, DMSO-*d*_*6*_): 11.86 (s, 1H, NH), 8.33−8.31 (m, 2H, triazole, CH), 7.95 (d, *J* = 8.2 Hz, 2H, H3′, H5′), 7.79−7.50 (m, 4H, H2, H3, H5, H6), 7.23−7.11 (m, 6H, H2′, H6′, H2′′, H3′′, H5′′, H6′′), 5.59 (s, 2H, CH_2_), 5.21 (s, 2H, CH_2_). ^13^CNMR (125 MHz, DMSO-*d*_*6*_): 164.1, 161.7 (d, *J*_*C−F*_ = 242.9 Hz), 159.9, 145.3, 142.0, 135.1, 133.0, 132.0, 130.1 (d, *J*_*C−F*_ = 7.8 Hz), 129.5, 128.8, 128.6, 124.8, 121.0, 115.4 (d, *J*_*C−F*_ = 22.9 Hz), 114.8, 61.1, 51.9 ppm. Calcd for C_24_H_19_ClFN_5_O_2_: C, 62.14; H, 4.13; N, 15.10. Found: C, 62.25; H, 4.09; N, 14.76.

#### 4-((1-(2-Chlorobenzyl)-1H-1,2,3-triazol-4-yl)methoxy)-*N*′-(4-chlorobenzylidene)benzohydrazide (7k)

Yellow precipitates, yield: 43%, mp 246−248 °C. IR (KBr, cm^−1^): 3416, 3049, 2923, 2852, 1688, 1631, 1591. ^1^HNMR (500 MHz, DMSO-*d*_*6*_): 11.82 (s, 1H, NH), 8.45−8.31 (m, 2H, CH, triazole), 8.22−7.91 (m, 2H, H3′, H5′), 7.76−7.51 (m, 5H, H2, H3, H5, H6, H3′′), 7.39- 7.17 (m, 5H, H2′, H6′, H4′′, H5′′, H6′′), 5.72 (s, 2H, CH_2_), 5.25 (s, 2H, CH_2_). ^13^CNMR (125 MHz, DMSO-*d*_*6*_): 162.2, 160.7, 147.4, 142.0, 134.5, 133.1, 132.6, 130.5, 130.2, 129.6, 129.2, 128.9, 128.8, 128.6, 127.7, 125.3, 120.5, 114.4, 61.1, 50.6 ppm. Calcd for C_24_H_19_Cl_2_N_5_O2: C, 60.01; H, 3.99; N, 14.58. Found: C, 60.19; H, 3.76; N, 14.45.

#### *N*′-(4-Chlorobenzylidene)-4-((1-(4-methylbenzyl)-1H-1,2,3-triazol-4-yl)methoxy)benzohydrazide (7l)

Red precipitates, yield: 40%, mp 216−218 °C. IR (KBr, cm^-1^): 3417, 3051, 2922, 2850, 1689, 1625, 1589. ^1^HNMR (500 MHz, DMSO-*d*_*6*_): 11.81 (s, 1H, NH), 8.60 (s, 1H, CH), 8.28 (s, 1H, triazole), 7.96- 7.54 (m, 6H, H2, H3, H5, H6, H3′, H5′), 7.21- 7.09 (m, 6H, H2′, H6′, H2′′, H3′′, H5′′, H6′′), 5.55 (s, 2H, CH_2_), 5.22 (s, 2H, CH_2_), 2.28 (s, 3H, CH3). ^13^CNMR (125 MHz, DMSO-*d*_*6*_): 161.8, 160.5, 147.5, 140.9, 137.4, 132.8, 132.0, 129.5, 129.2, 128.8, 128.5, 127.9, 125.8, 123.7, 120.9, 114.8, 62.0, 52.6, 20.6 ppm. Calcd for C_25_H_22_ClN_5_O_2_: C, 65.29; H, 4.82; N, 15.23. Found: C, 65.63; H, 4.63; N, 15.19.

### 4-((1-Benzyl-1H-1,2,3-triazol-4-yl)methoxy)-*N*′-(4-methoxybenzylidene)benzohydrazide (7m)

Yellow precipitates, yield: 53%, mp 237−240 °C. IR (KBr, cm^−1^): 3415, 3053, 2921, 2852, 1686, 1633, 1589. ^1^HNMR (500 MHz, DMSO-*d*_*6*_): 11.86 (s, 1H, NH), 8.31−8.30 (m, 2H, triazole, CH), 7.88−7.71 (m, 4H, H2, H6, H3′, H5′), 7.36−7.32 (m, 5H, H2′′, H3′′ H4′′, H5′′, H6′′), 7.15−7.01 (m, 4H, H3, H5, H2′, H6′), 5.56 (s, 2H, CH_2_), 5.21 (s, 2H, CH_2_), 3.78 (s, 3H, OCH3). ^13^CNMR (125 MHz, DMSO-*d*_*6*_): 162.0, 161.5, 158.8, 147.5, 142.8, 135.9, 130.3, 128.7, 128.1, 127.9, 127.6, 126.4, 125.5, 120.2, 114.4, 113.8, 61.2, 55.2, 52.8 ppm. Calcd for C_25_H_23_N_5_O_3_: C, 68.01; H, 5.25; N, 15.86. Found: C, 68.1; H, 5.42; N, 15.81.

### 4-((1-(4-Fluorobenzyl)-1H-1,2,3-triazol-4-yl)methoxy)-*N*′-(4-ethoxybenzylidene)benzohydrazide (7n)

Light green precipitates, yield: 58%, mp 244−247 °C. IR (KBr, cm^−1^): 3416, 3051, 2926, 2852, 1685, 1633, 1591. ^1^HNMR (500 MHz, DMSO-*d*_*6*_): 11.63 (s, 1H, NH), 8.40 (s, 1H, CH), 8.32 (s, 1H, triazole), 7.89 (d, *J* = 8.3 Hz, 2H, H3′, H5′), 7.41−7.20 (m, 6H, H2, H6, H2′′, H3′′, H5′′, H6′′), 7.15 (d, *J* = 8.3 Hz, 2H, H2′, H6′), 7.02 (d, *J* = 8.2 Hz, 2H, H3, H5), 5.62 (s, 2H, CH_2_), 5.23 (s, 2H, CH_2_), 3.81 (s, 3H, OCH_3_). ^13^CNMR (125 MHz, DMSO-*d*_*6*_): 162.8, 161.3, 161.2 (d, *J*_*C−F*_ = 245.0 Hz), 157.0, 147.1, 142.0, 132.2, 130.3 (d, *J*_*C−F*_ = 8.2 Hz), 129.8, 129.4, 128.7, 124.8, 122.5, 115.6 (d, *J*_*C−F*_ = 21.6 Hz), 114.4, 114.3, 61.2, 55.3, 52.1 ppm. Calcd for C_25_H_22_FN_5_O_3_: C, 65.35; H, 4.83; N, 15.24. Found: C, 65.57; H, 5.21; N, 15.13.

### 4-((1-(2-Chlorobenzyl)-1H-1,2,3-triazol-4-yl)methoxy)-*N*′-(4-methoxybenzylidene)benzohydrazide (7o)

Green precipitates, yield: 43%, mp 222−225 °C. IR (KBr, cm^−1^): 3417, 3050, 2926, 2850, 1688, 1637, 1591. ^1^HNMR (500 MHz, DMSO-*d*_*6*_): 11.61 (s, 1H, NH), 8.30−8.29 (m, 2H, triazole, CH), 7.94−7.78 (m, 5H, H2, H6, H3′, H5′, H3′′), 7.24−7.16 (m, 5H, H2′, H6′, H4′′, H5′′, H6′′), 7.07 (d, *J* = 8.1 Hz, 2H, H3, H5), 5.73 (s, 2H, CH_2_), 5.24 (s, 2H, CH_2_), 3.81 (s, 3H, OCH_3_). ^13^CNMR (125 MHz, DMSO-*d*_*6*_): 163.0, 160.6, 159.0, 144.9, 142.5, 133.1, 132.6, 130.5, 130.2, 129.6, 129.4, 128.6, 127.7, 126.8, 126.2, 121.1, 114.3, 113.5, 61.2, 55.2, 50.6 ppm. Calcd for C_25_H_22_ClN_5_O_3_: C, 63.09; H, 4.66; N, 14.72. Found: C, 63.22; H, 4.39; N, 14.87.

### *N*′-(4-Methoxybenzylidene)-4-((1-(4-methylbenzyl)-1H-1,2,3-triazol-4-yl)methoxy)benzohydrazide (7p)

Light brown precipitates, yield: 57%, mp 224−228 °C. IR (KBr, cm^−1^): 3415, 3052, 2927, 2850, 1687, 1630, 1587. ^1^HNMR (500 MHz, DMSO-*d*_*6*_): 11.68 (s, 1H, NH), 8.26−8.25 (m, 2H, triazole, CH), 7.88−7.69 (m, 4H, H2, H6, H3′, H5′), 7.21−7.02 (m, 8H, H3, H5, H2′, H6′, H2′′, H3′′, H5′′, H6′′), 5.54 (s, 2H, CH_2_), 5.19 (s, 2H, CH_2_), 3.79 (s, 3H, OCH_3_), 2.28 (s, 3H, CH3). ^13^CNMR (125 MHz, DMSO-*d*_*6*_): 161.9, 160.8, 159.3, 146.0, 144.0, 137.5, 132.9, 130.0, 129.2, 128.9, 128.5, 128.0, 125.5, 121.0, 114.1, 113.9, 61.2, 54.8, 52.6, 20.6 ppm. Calcd for C_26_H_25_N_5_O_3_: C, 68.56; H, 5.53; N, 15.37. Found: C, 68.35; H, 5.61; N, 15.53.

### In vitro α-glucosidase inhibition assay

The assay was performed exactly according to our previous report^30,38^.

### Enzyme kinetic studies

The mode of inhibition of the most active compounds **7a** and **7h** (identified with the lowest IC_50_) was investigated against α-glucosidase activity with different concentrations of *p*-nitrophenyl *α*-D-glucopyranoside (1−10 mM) as substrate in the absence and presence of inhibitors at different concentrations. **7h**: 0, 2, 4, and 8 nM and **7a**: 0, 0.2, 0.5, and 1 µM.

### Fluorescence spectroscopy

The fluorescence measurements were performed on a Synergy HTX multi-mode reader (Biotek Instruments, Winooski, VT, USA) equipped with a quartz cuvette of 10 mm. The excitation wavelength was 280 nm and the emission spectra were measured in the range from 300 to 440 nm with 10 accumulations for each collection point. The emission spectrum was corrected for the background fluorescence from the buffer solution and for the inner filter effect promoted by the inhibitors. The protein-inhibitors interaction measurements were performed by collecting the fluorescence.

The fluorescence quenching of the protein (*P*) by a drug or inhibitor (*D*) was analyzed by Stern−Volmer equation (Eq. 1), where *F*_0_ and *F* are the fluorescence intensity in the absence and presence of a quencher (inhibitor), respectively. [*D*] is the concentration of inhibitor, *K*_q_ is the quenching rate constant, τ_0_ is the average life-time of the molecule without the quencher (10^−8^ s), and *K*_SV_ is the Stern−Volmer dynamic quenching constant.

1 $$F_{0} /F = \, 1 \, + K_{q} \tau_{0} \left[ D \right] \, = \, 1 \, + K_{SV} \left[ D \right]$$

The reaction of the protein (*P*) and a drug molecule or inhibitor (*D*) was considered as Eq. 2, where *P* is the protein, *D* is the drug molecule, and *D*_*n*_*P* is the new complex molecule with the binding constant of *K*_A_.

2 $$P + D \to D_{n} P$$

As the number of the binding site of protein and drug is respectively* n* and 1, the equivalent concentration of the complex *D*_*n*_*P* is *n*[*D*_*n*_*P*]; the equivalent concentration of the protein is *n*[*P*] and the equivalent concentration of the drug is [*D*]. In this respect, the *K*_A_ value is calculated based on the Eq. (3).

3 $$K_{A} = n\left[ {D_{n} P} \right]/\left[ D \right]n\left[ P \right]$$

If the total concentration of protein is [*P*_t_] and the total concentration of the drug is [*D*_t_]: [*P*_t_] = [*P*_f_] + [D_*n*_P] and [*D*_f_] = [*D*_t_] − *n*[D_*n*_P].

If protein (*P*) is the only fluorescence in the reaction system, thus:

4 $$F_{0} /F = \, \left[ {P_{t} } \right]/\left[ {P_{f} } \right]$$

where *F* and *F*_0_ are the fluorescence intensity of protein in the presence and absence of drug *D*, respectively. Therefore, the relationship between the fluorescence intensity and the drug total concentration can be deduced from Eq. (5).

5 $$F_{0} /F = K_{A} \left[ {D_{t} } \right]F_{0} /\left( {F_{0} {-}_{{}} F} \right) \, {-}n \, K_{A} \left[ {P_{t} } \right]$$

Keeping the total concentration of protein at a definite value and varying the total concentration of the drug leads to obtaining the plot of *F*_0_/*F* as a function of [*D*_t_] *F*_0_/(*F*_0_ − *F*) following with the calculation of *K*_A_.

The standard thermodynamic values were calculated according to Eqs. (6), (7), where Δ*H*, Δ*G*, and Δ*S* are enthalpy, free energy, and entropy changes, respectively. In the case of small changes in temperature, the enthalpy change can be considered as a constant and Δ*H* is calculated from Eq. (6) following with the calculation of Δ*G* and Δ*S* from Eq. (7).

6 $$\ln K_{A2} /K_{A1} = \, \left( {1/T_{1} - 1/T_{2} } \right) \, \Delta H/R$$

7 $$\Delta G = \, {-}RT\ln K_{A} = \, \Delta H{-}T\Delta S$$

### Molecular docking procedure

To prepare the ligands, the 2D structures of the ligands were drawn in ChemDraw (ver. 16), converted into SDF and software, and energy was minimized by the MM1 force field. The docking simulation was performed on the crystal structures of human lysosomal acid-alpha-glucosidase (PDB ID: 5NN8) to figure out the binding modes of the selected compounds. The crystal structures of acarbose were retrieved from the PDB and docked using the Molegro Virtual Docker software. The site map was tasked to report up to 5 potential binding sites with at least 15 site points per each reported site by a more restrictive definition of hydrophobicity. Flexible ligand dockings were accomplished for the selected compounds with the scoring function of MolDock Score, Grid resolution of 0.30, Radios 10, search algorithm of Moldock Se, Max population size of 100, and max iteration 1500. The best pose of the selected compound in the target protein was chosen by analyzing the interactions between the enzyme and inhibitors. The best-scoring positions, as achieved by the docking score, were then selected, and visualized using Discovery Studio Client 2017.

## Supplementary Information

Supplementary Information.

## Data Availability

All data generated or analyzed during this study are included in this published article and its supplementary information files.
