# Supplementary material for: Novel N′-substituted benzylidene benzohydrazides linked to 1,2,3-triazoles: potent α-glucosidase inhibitors
Source: Sci Rep. 2023 Jun 2;13:8960. doi: 10.1038/s41598-023-36046-y (PMC10235848; doi:10.1038/s41598-023-36046-y)

**Supplementary file**

***Novel N'-substituted benzylidene benzohydrazides linked to 1,2,3-triazoles: potent α-glucosidase inhibitors***

**Compound 7b**


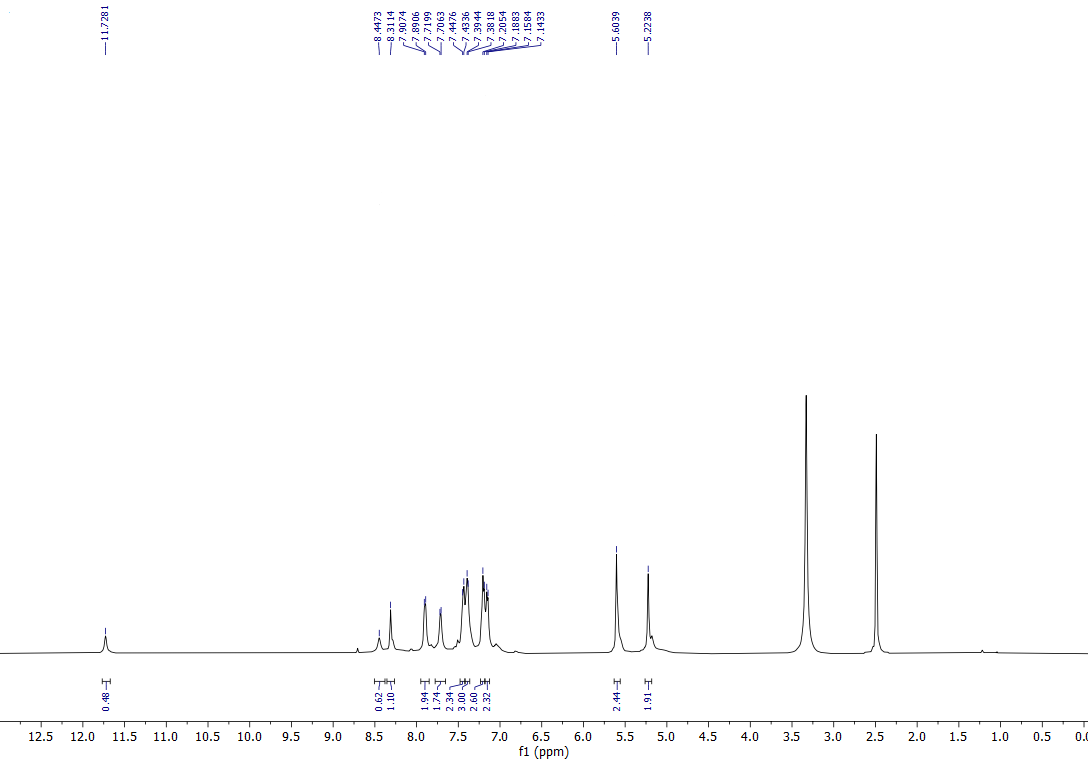


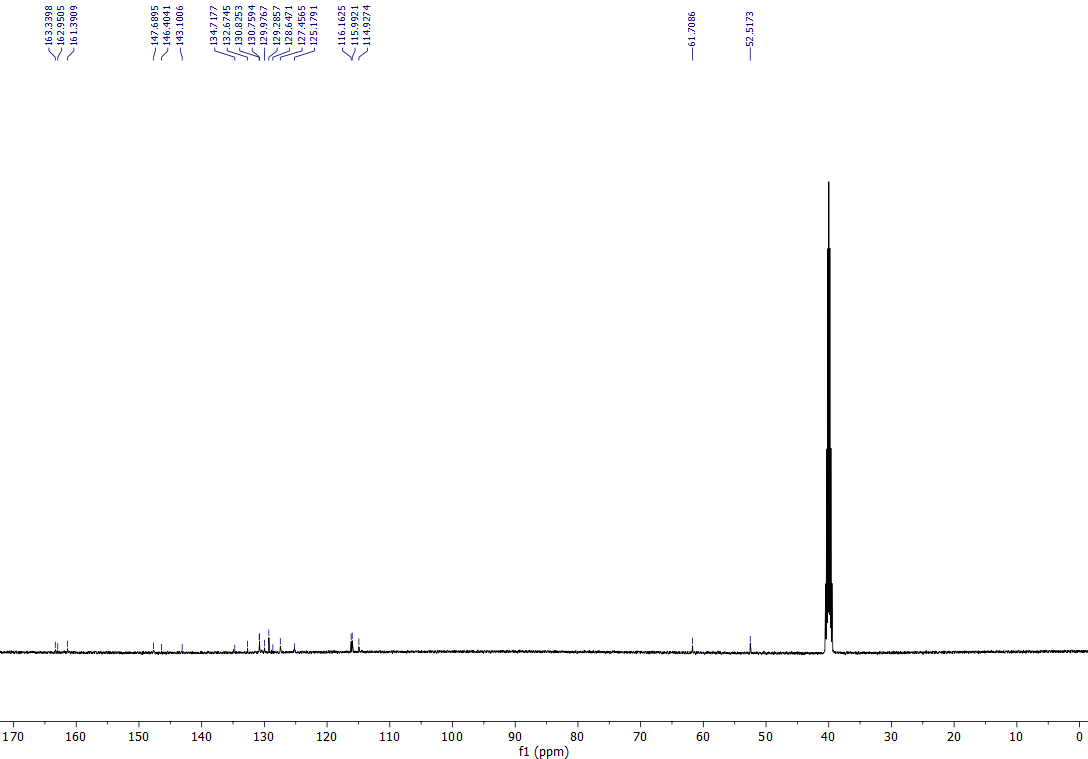


**Compound 7c**


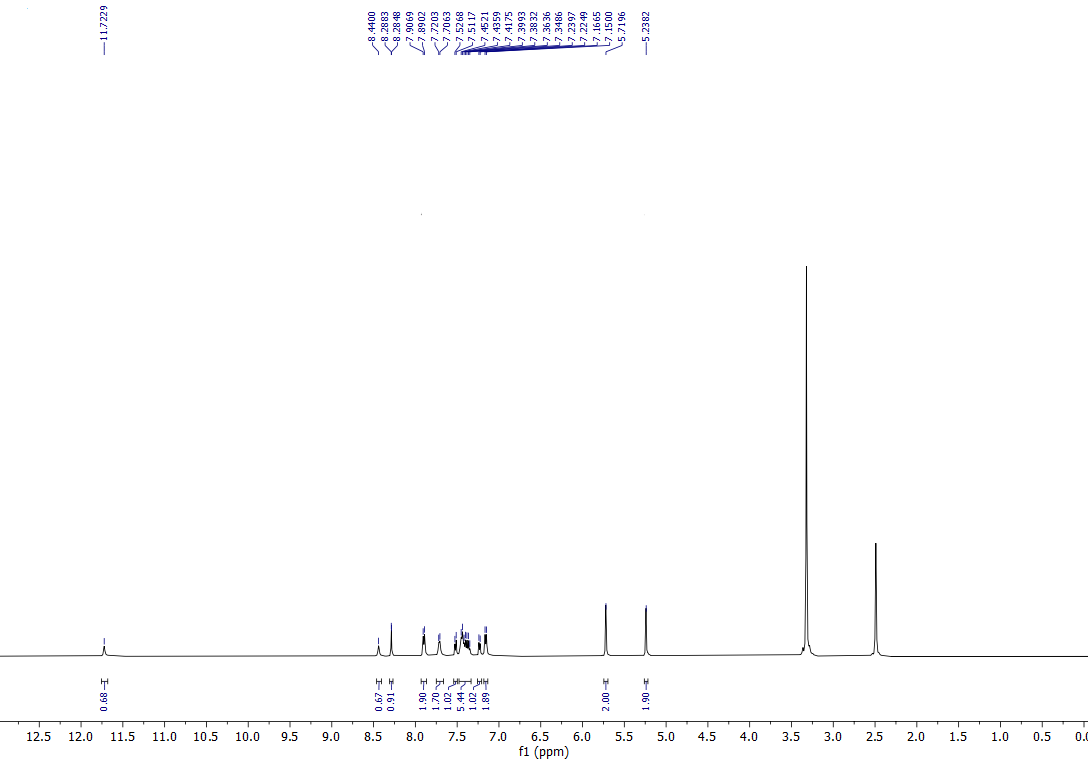


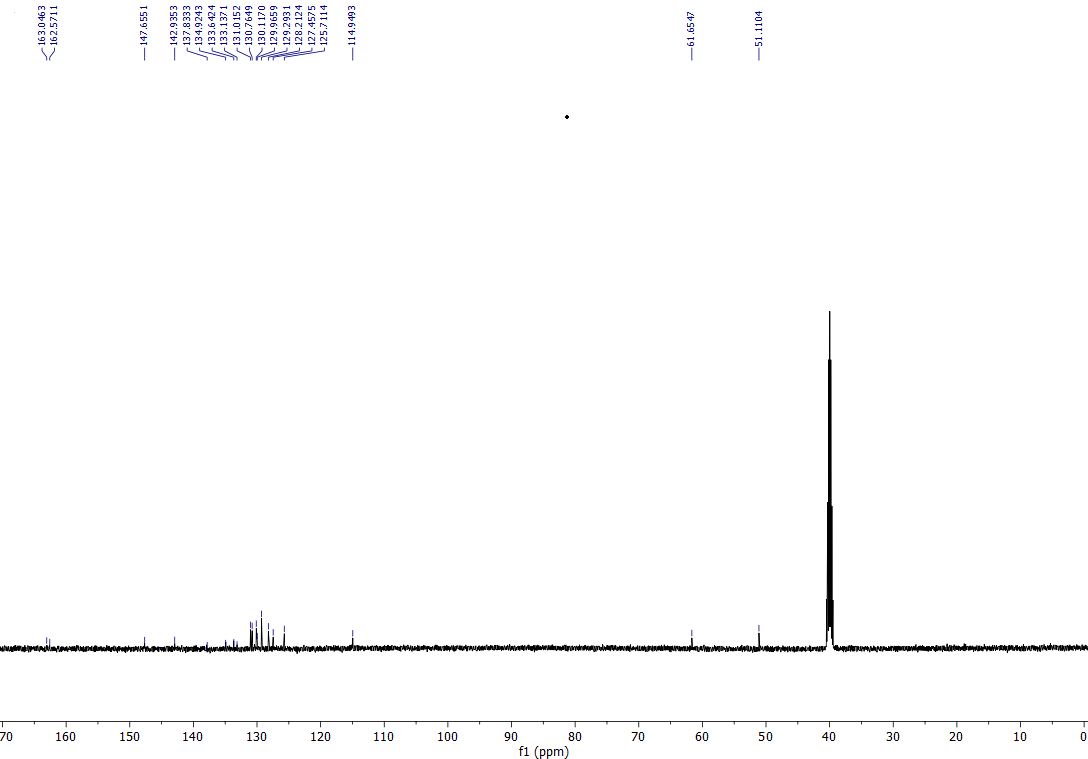


**Compound 7d**


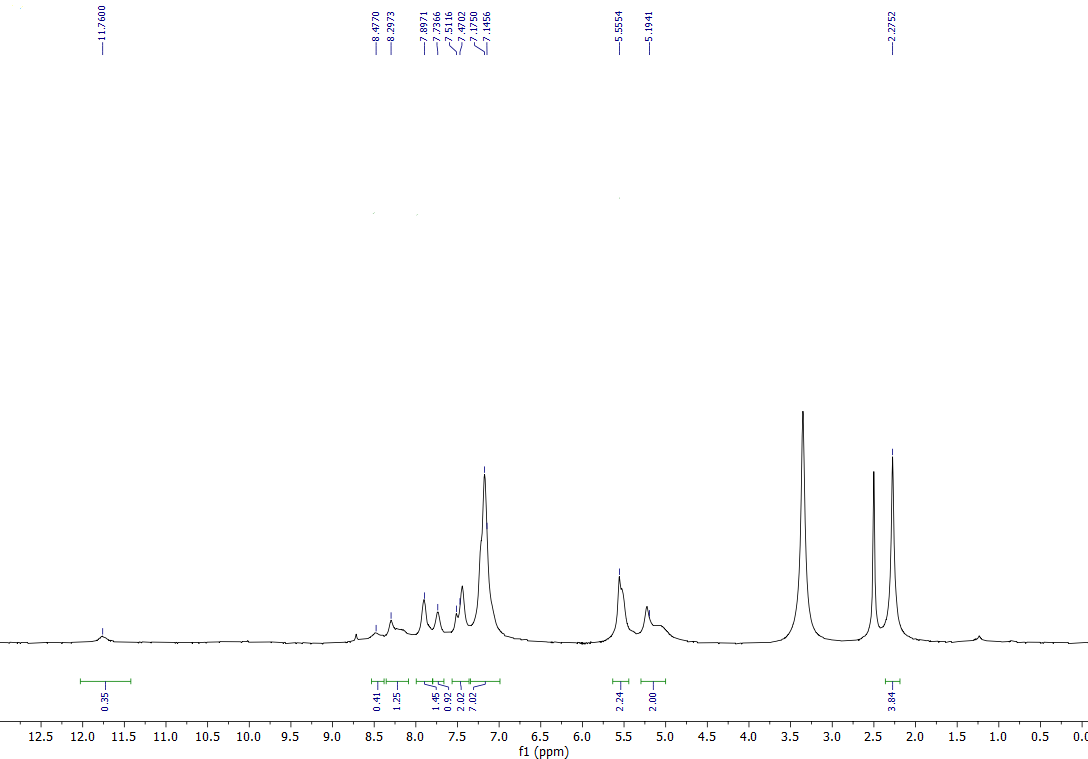


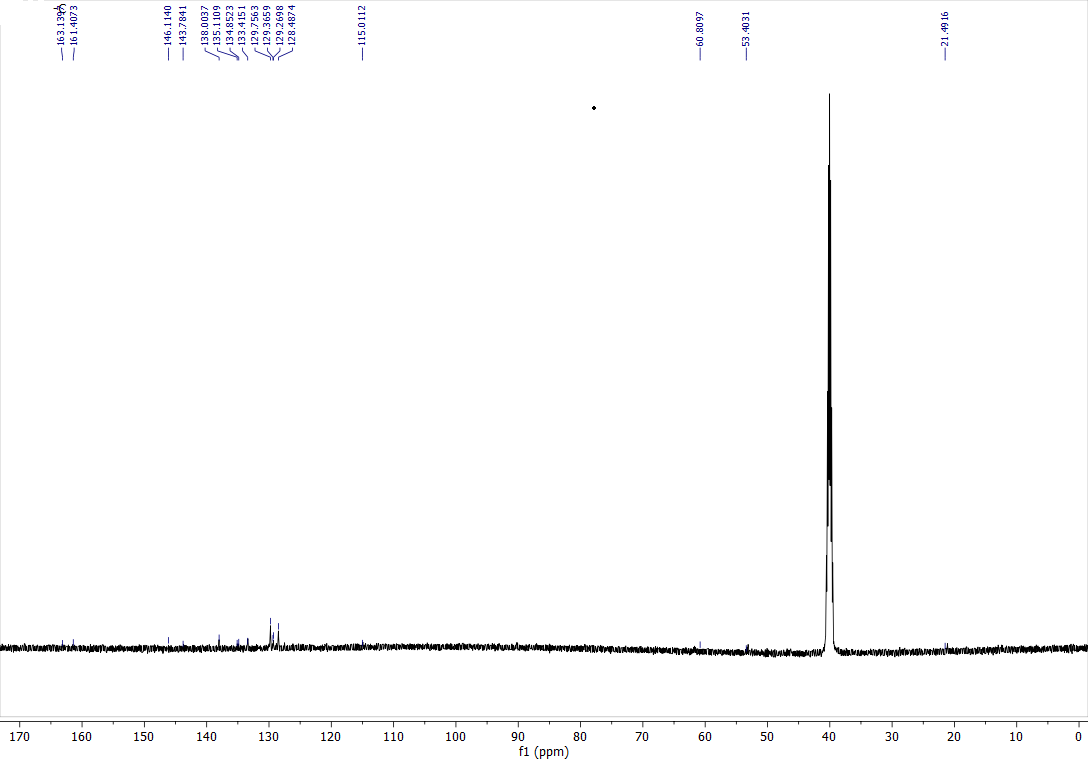


**Compound 7f**


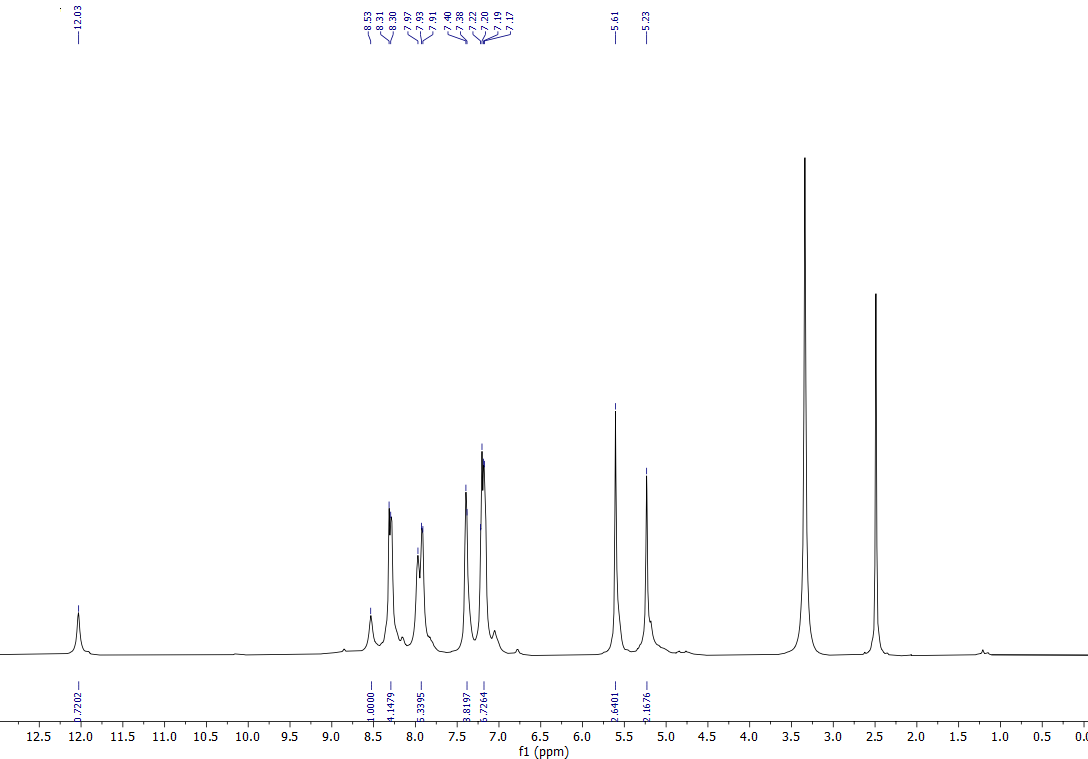


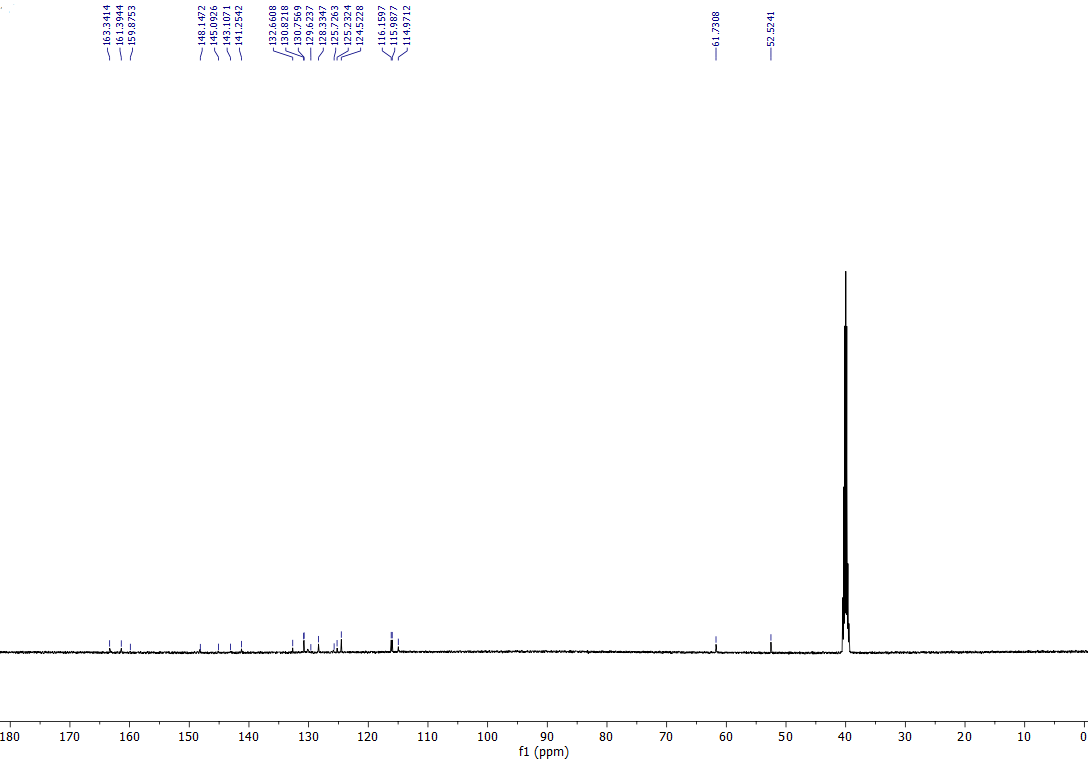


**Compound 7g**


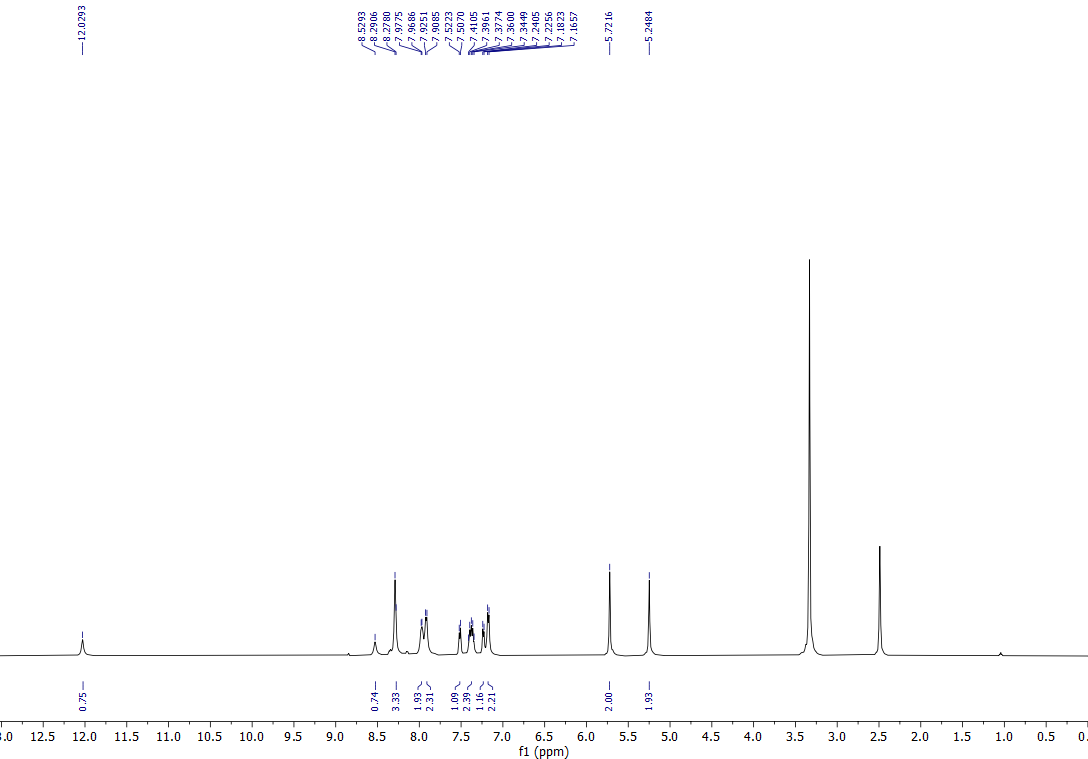


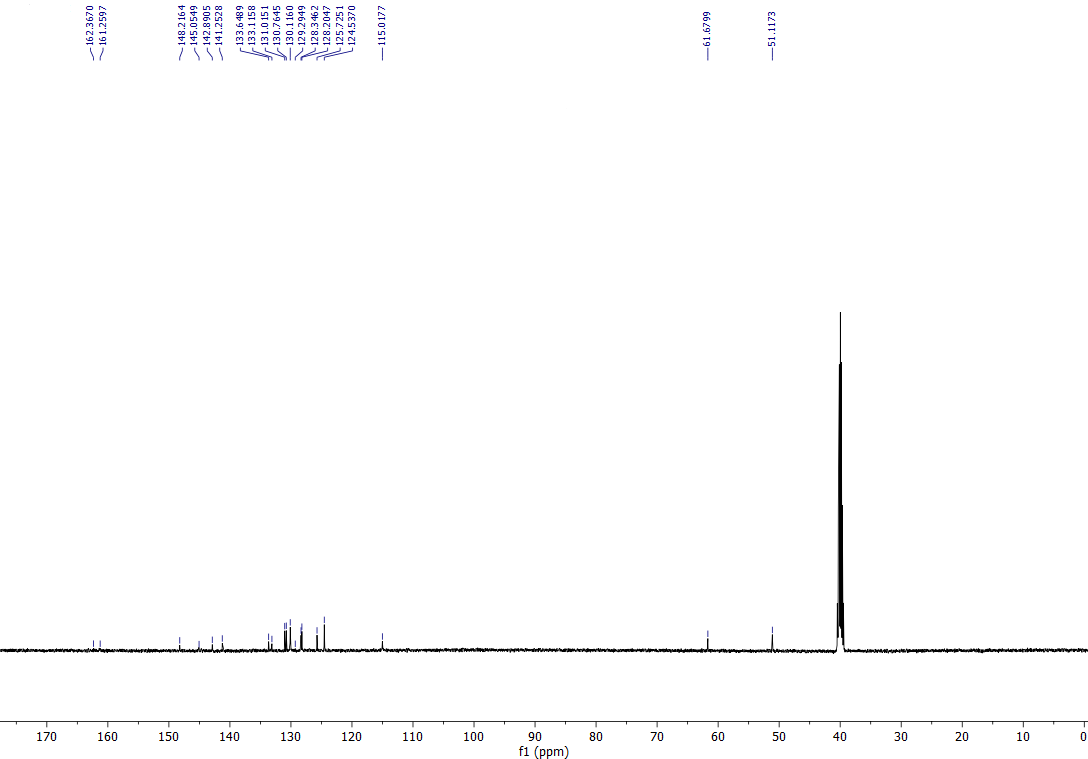


**Compound 7h**
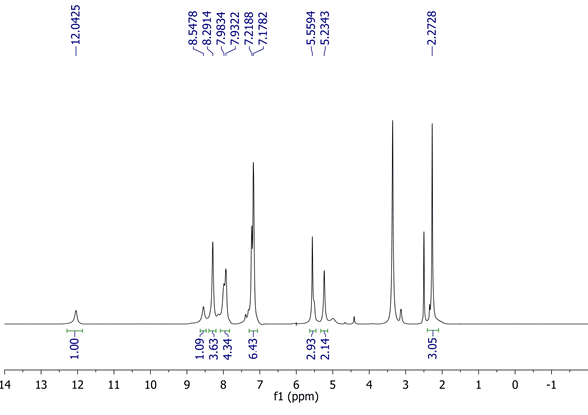


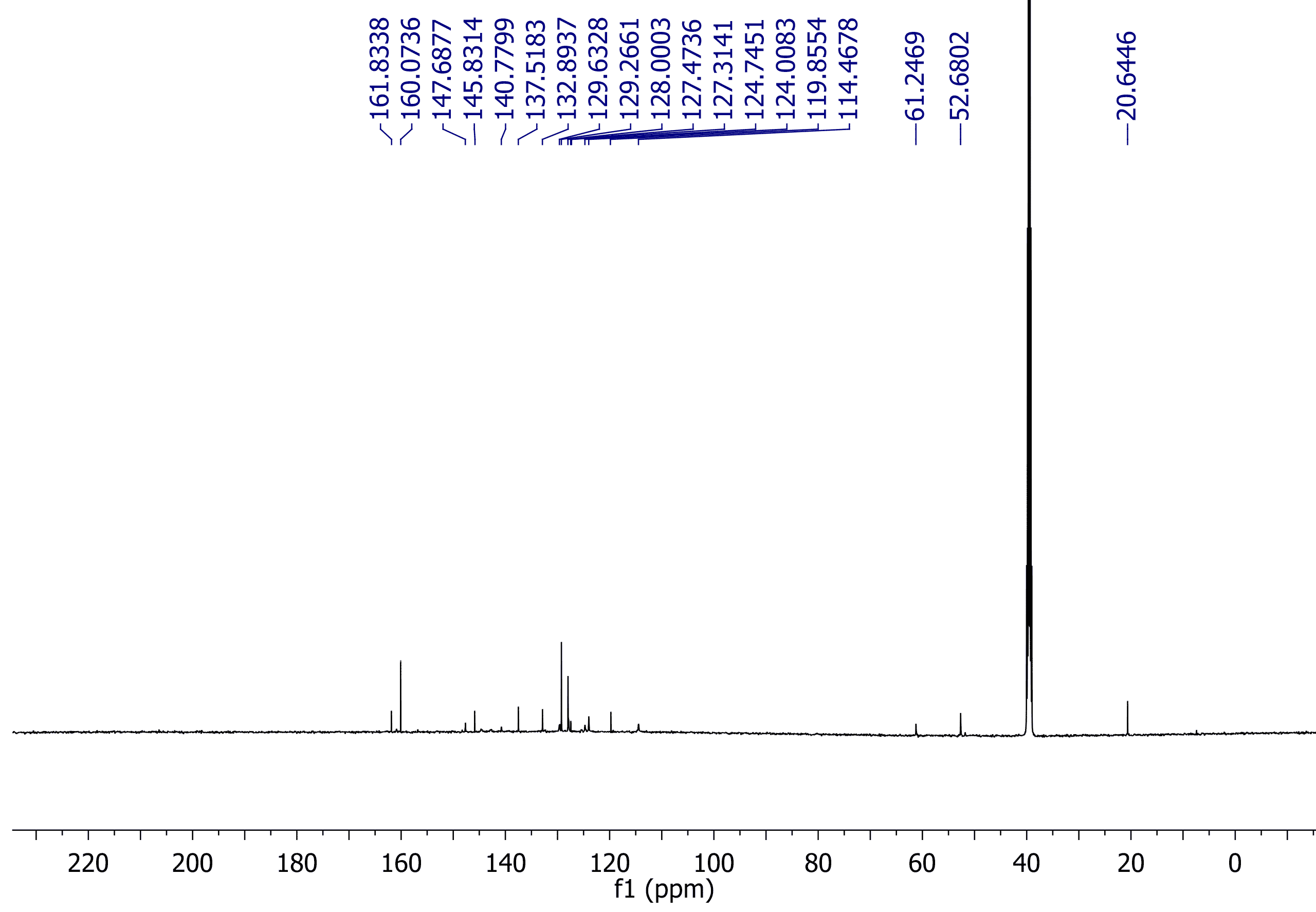


**Compound 7i**
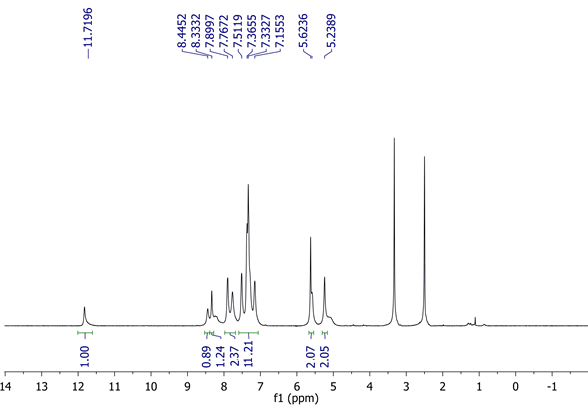


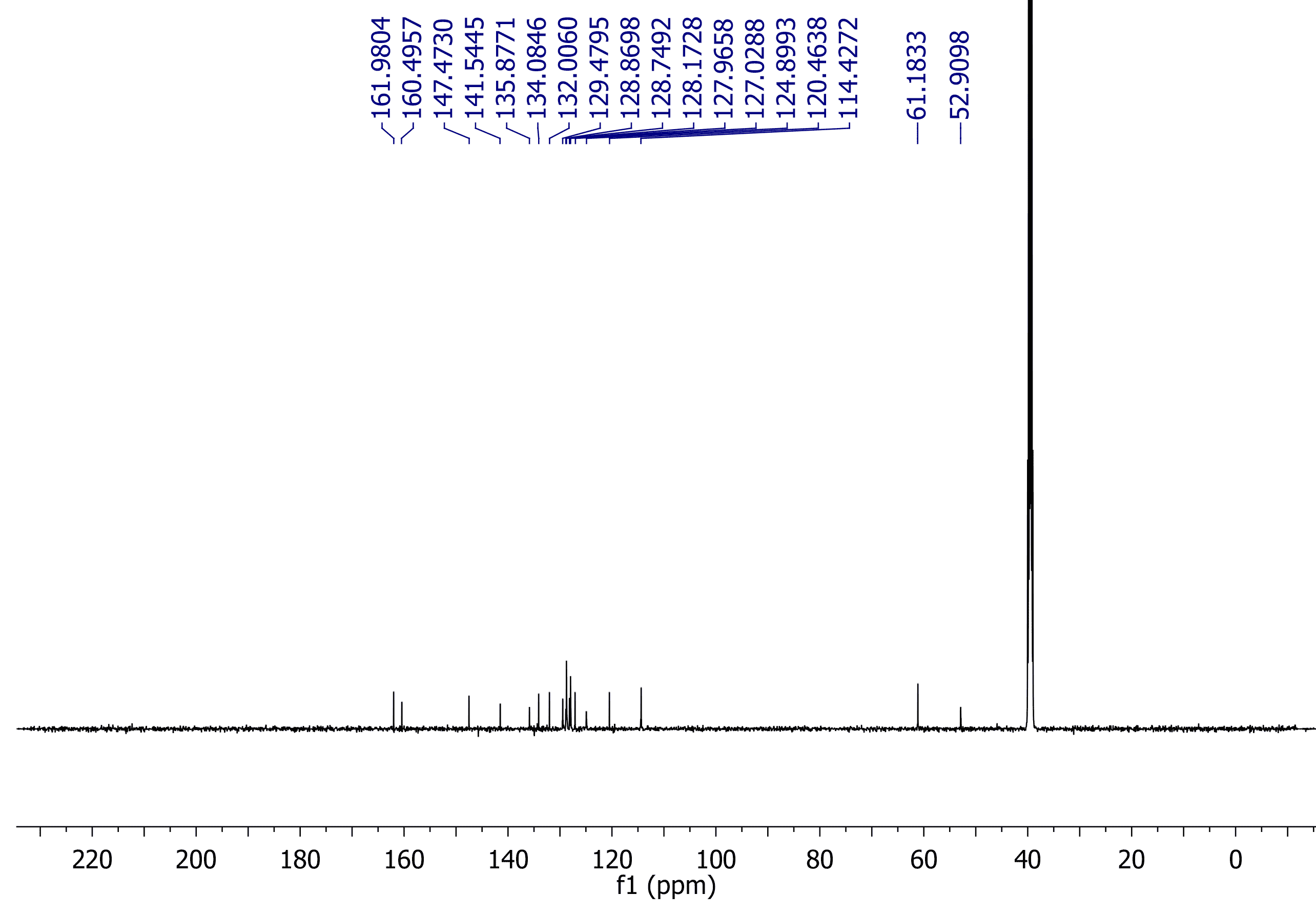


**Compound 7j***
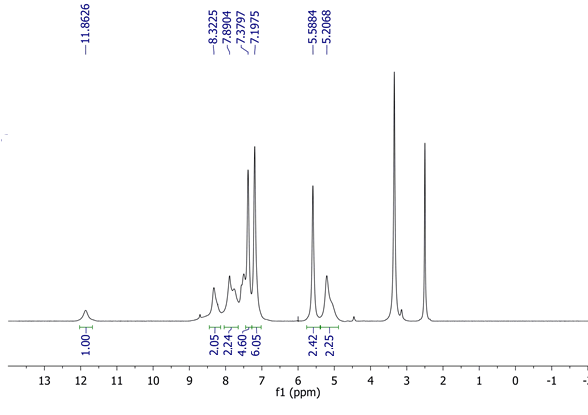
*


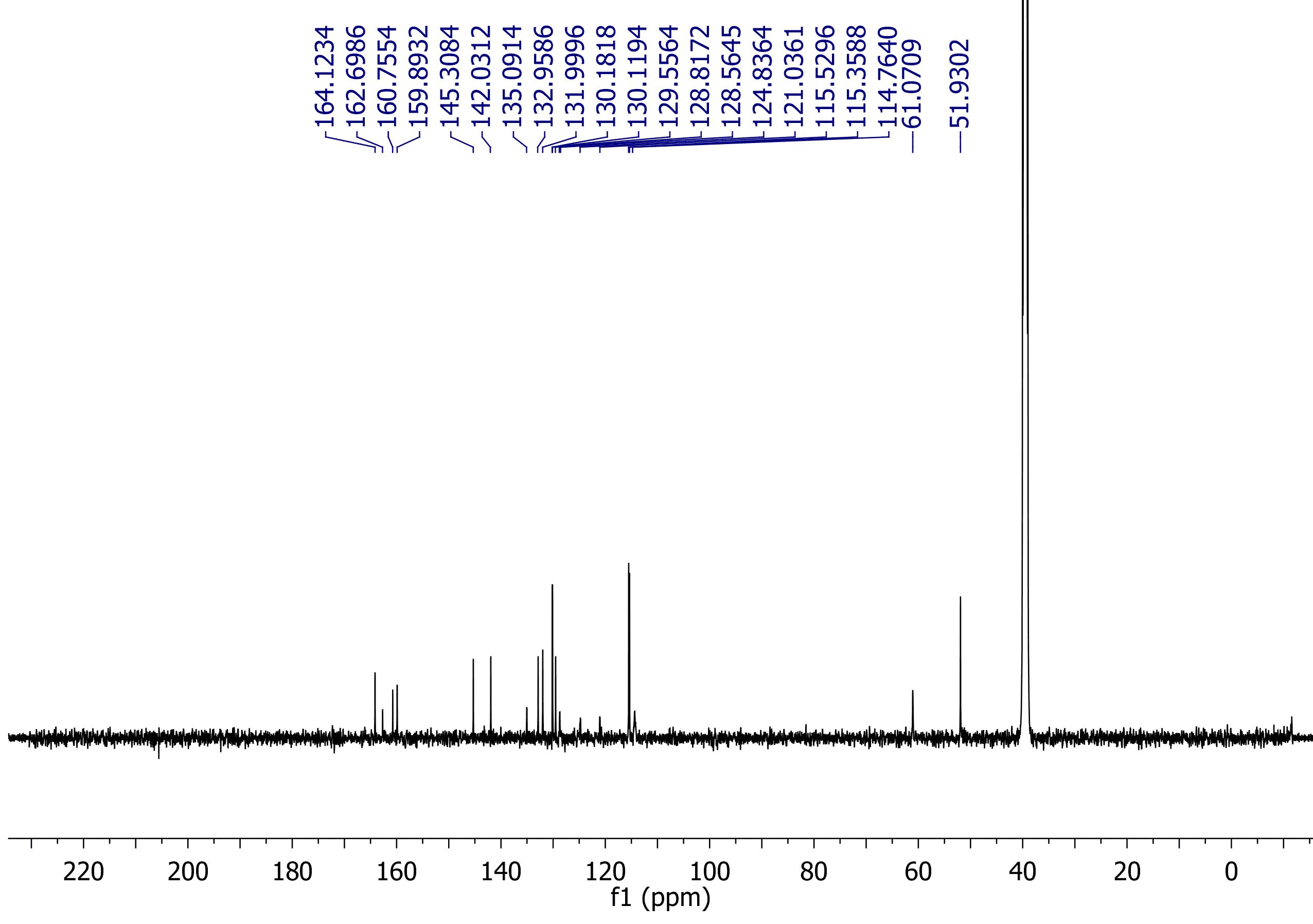


**Compound 7k**
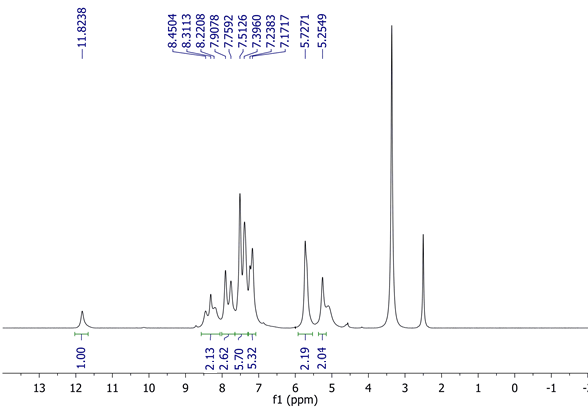


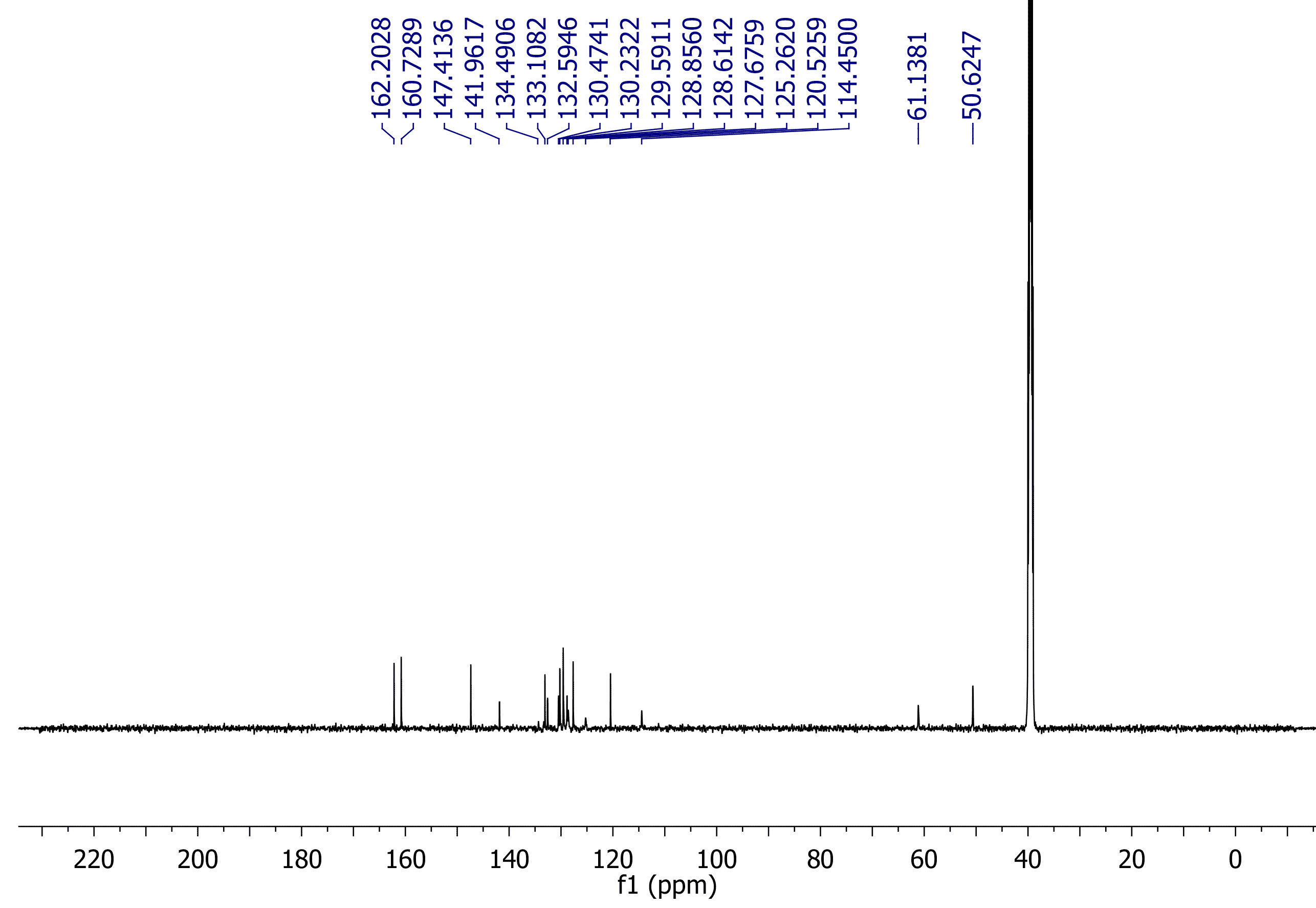


**Compound 7l**

*
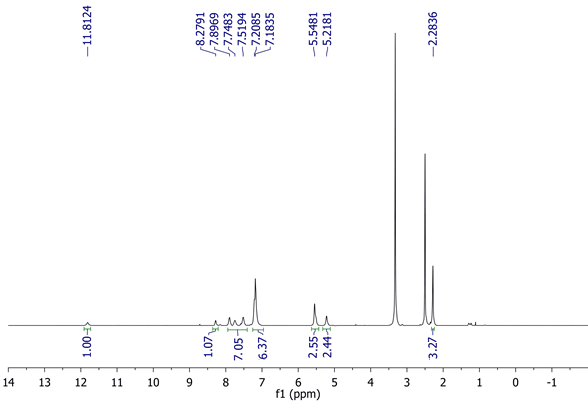
*


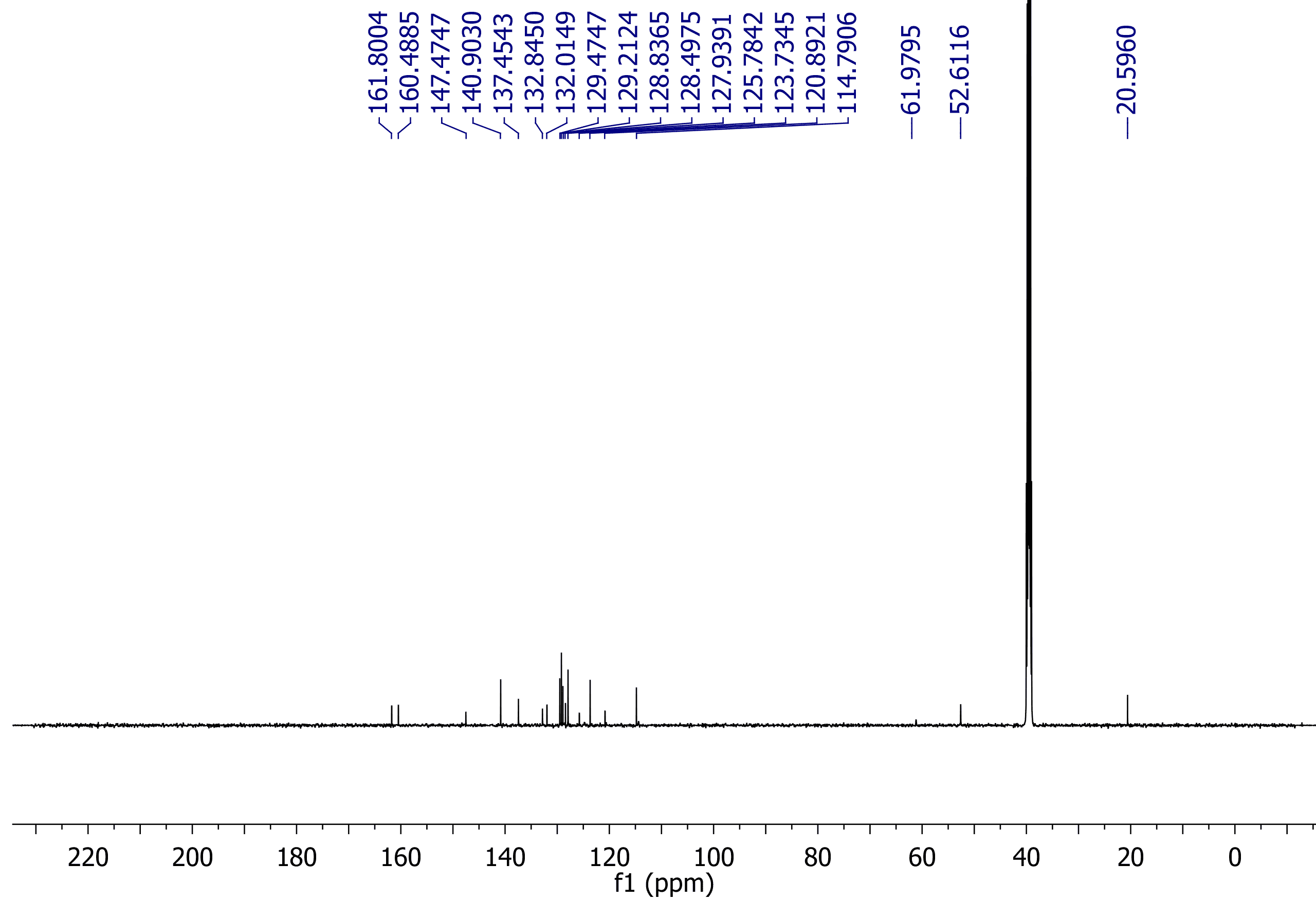


**Compound 7m**

*
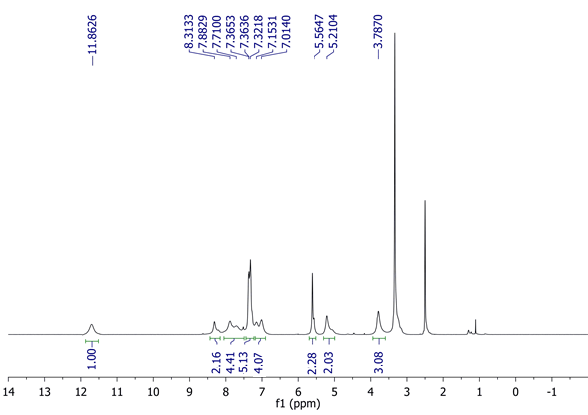
*


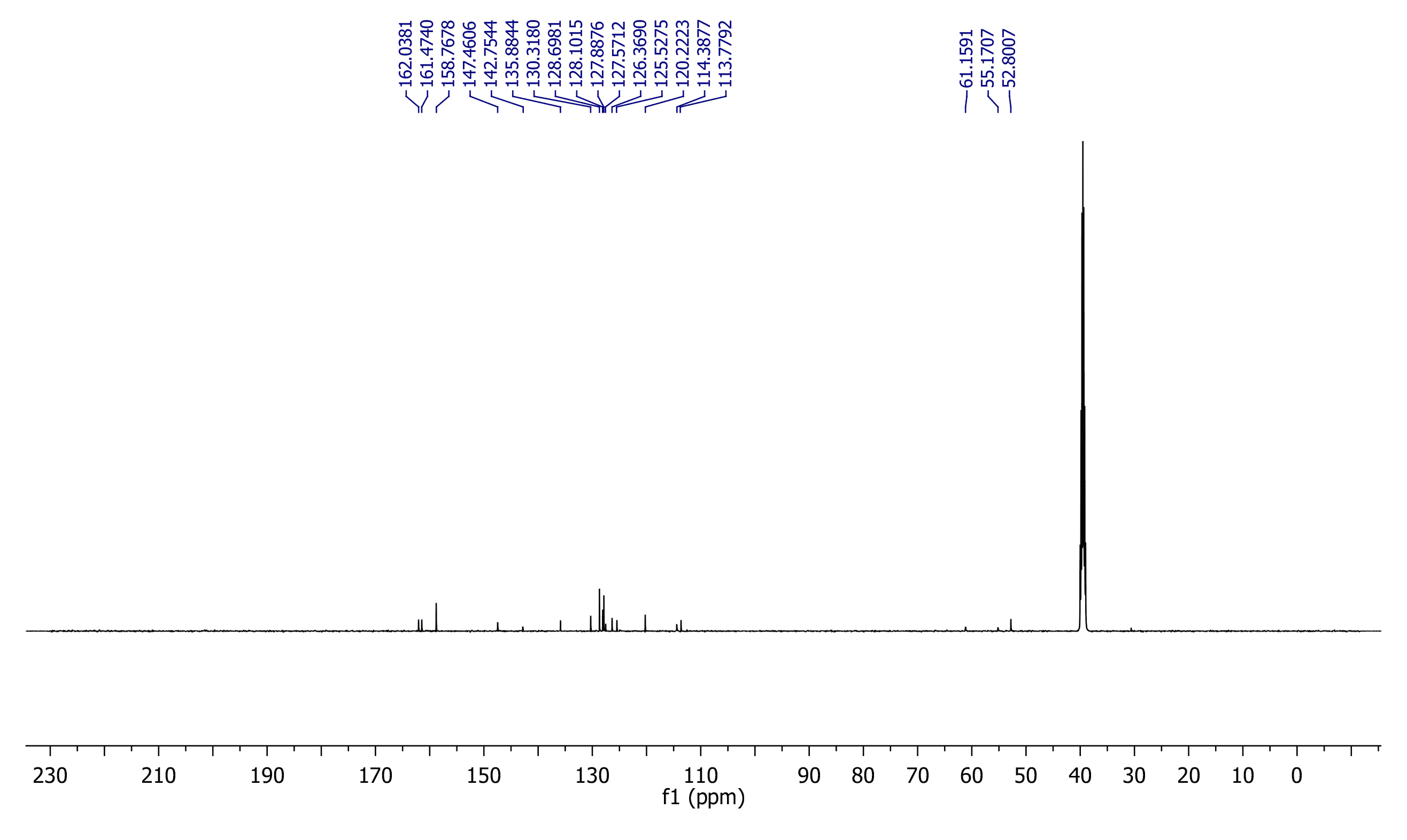


**Compound 7n**

*
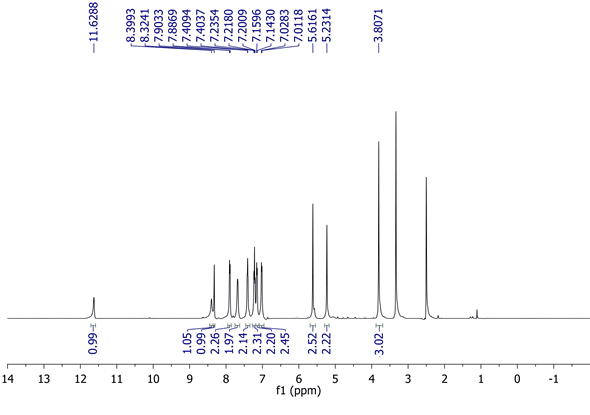
*


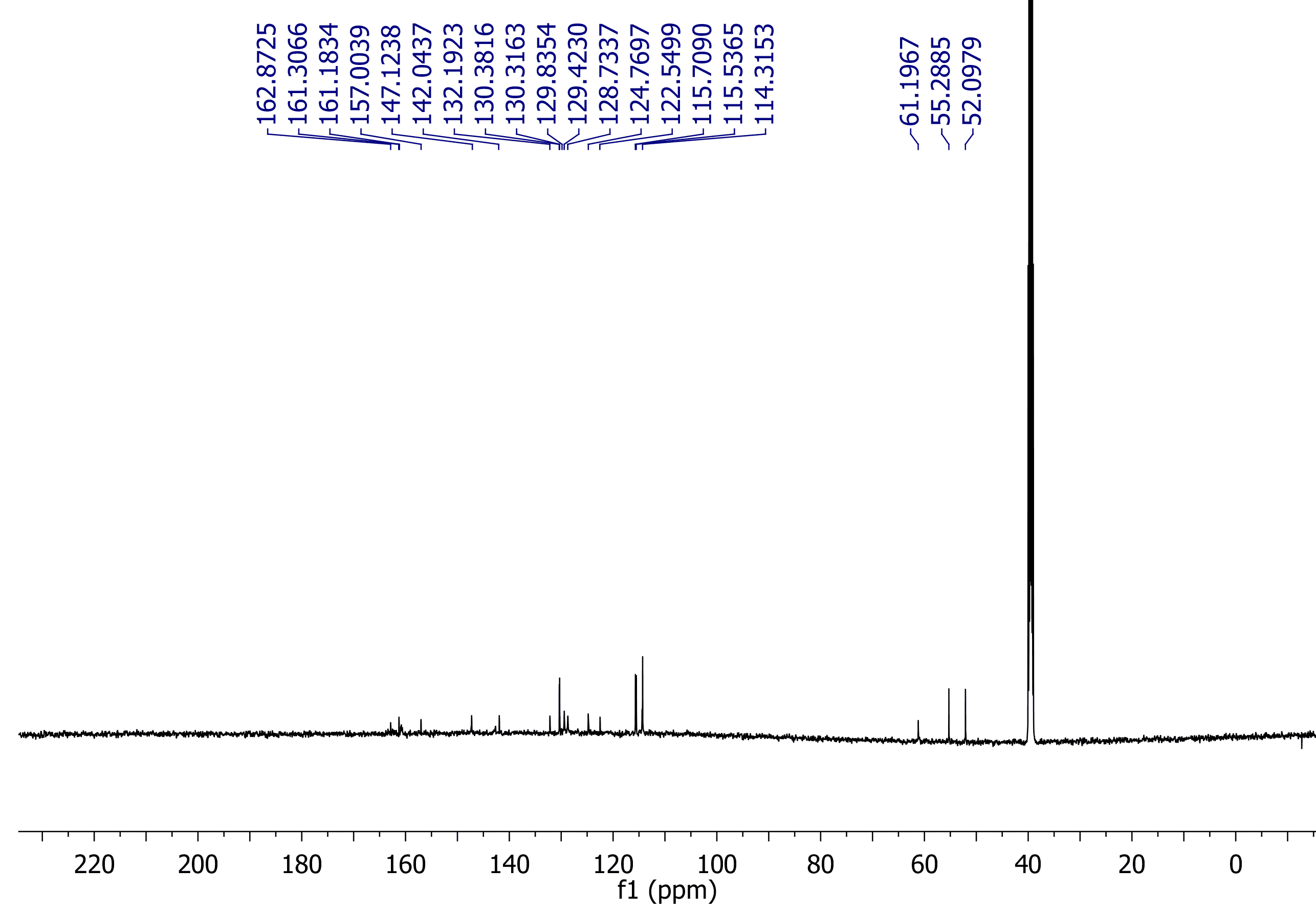


**Compound 7o**

*
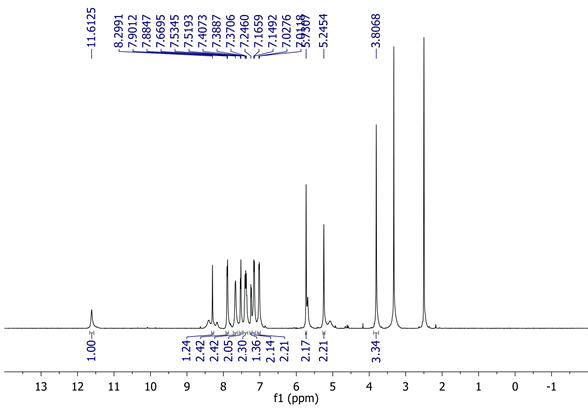
*


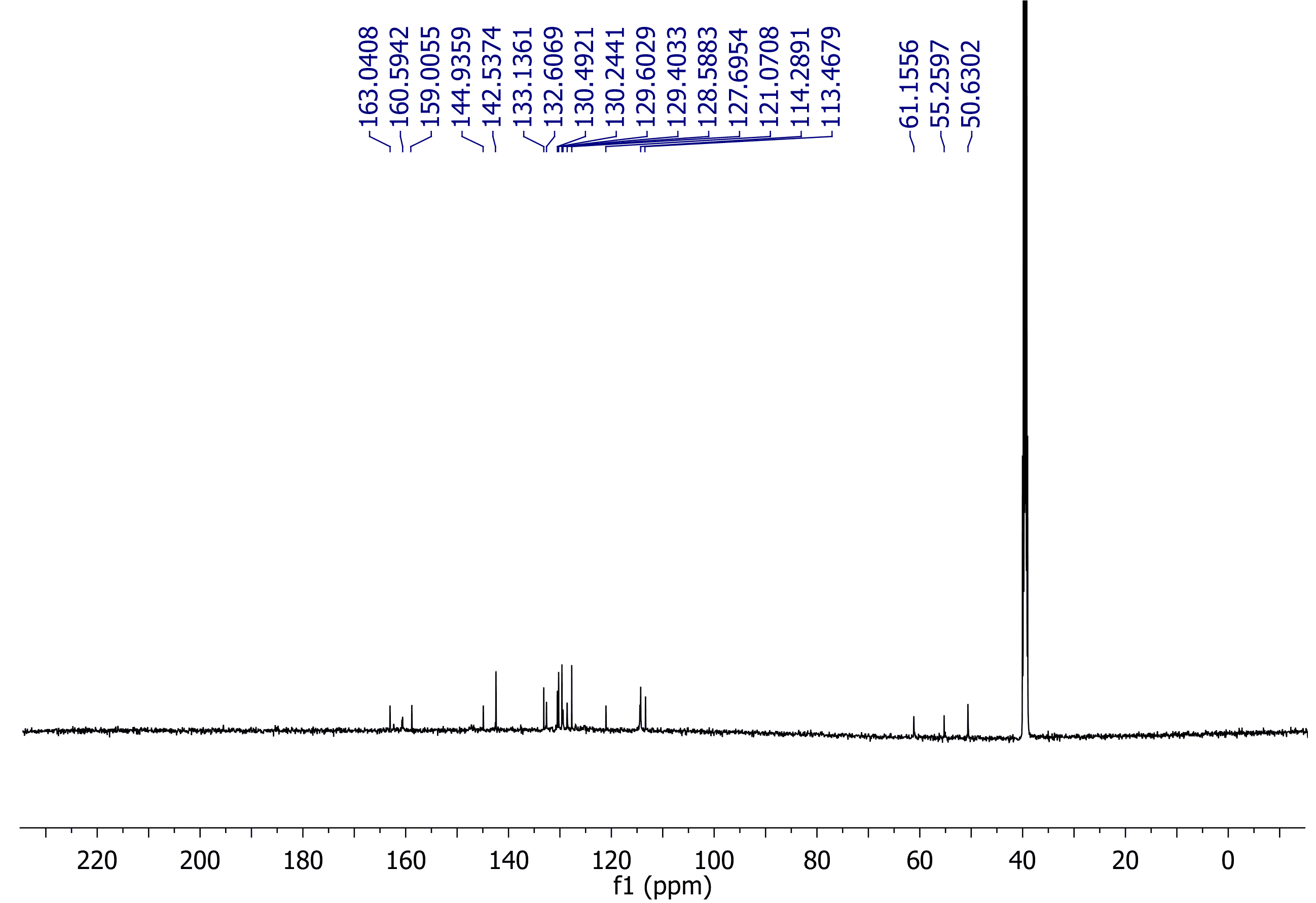


**Compound 7p**
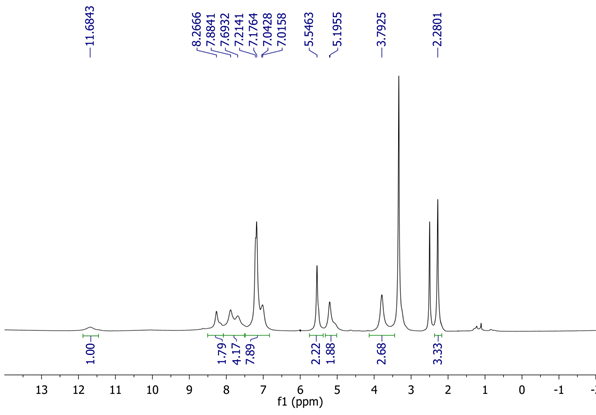


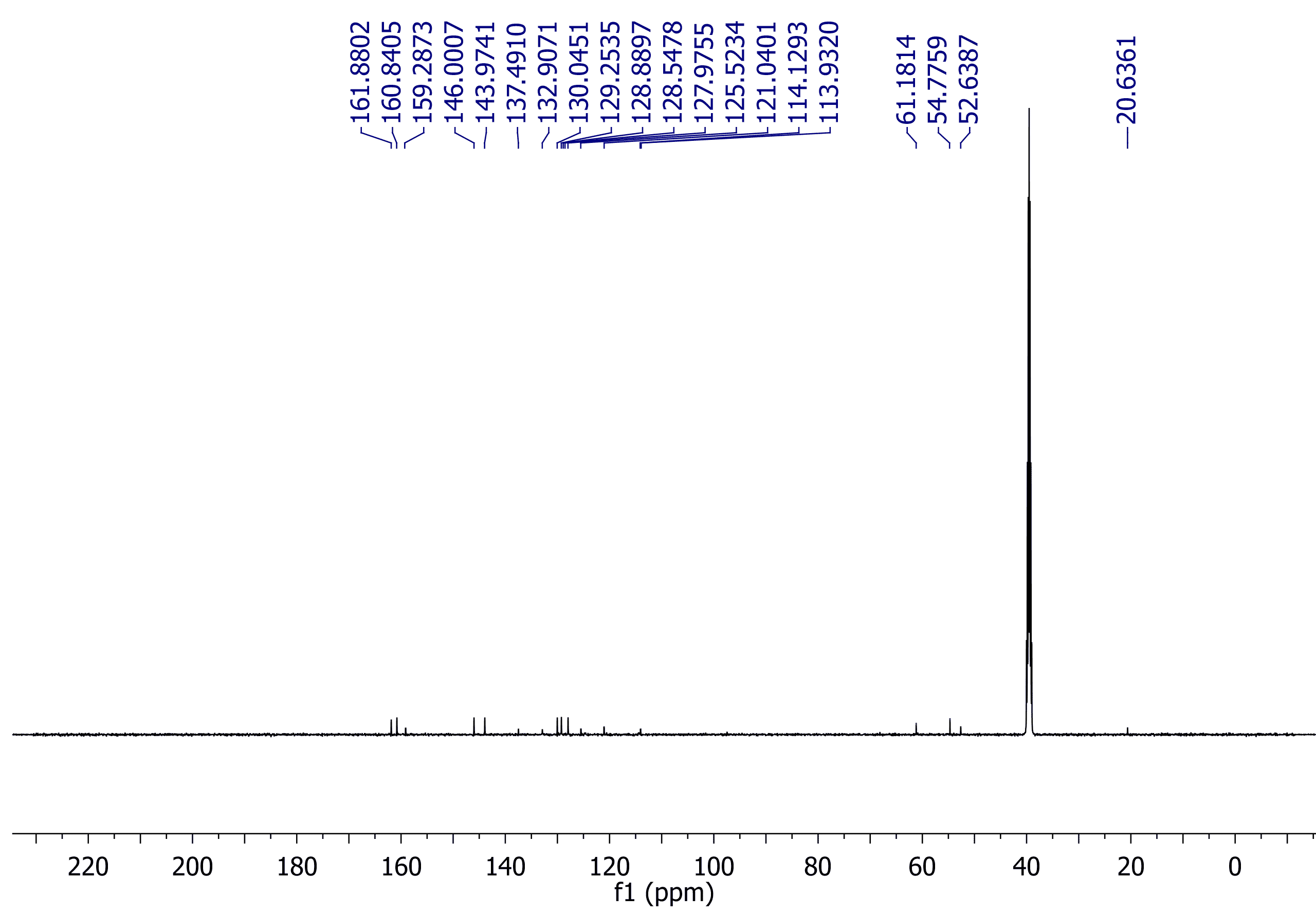

Supplement: Supplementary file 1 — Supplementary Information. [file 41598_2023_36046_MOESM1_ESM.docx]
